# Dual redox-active porous polyimides as high performance and versatile electrode material for next-generation batteries†

**DOI:** 10.1039/d2mh01335e

**Published:** 2023-01-09

**Authors:** Nicolas Goujon, Marianne Lahnsteiner, Daniel A. Cerrón-Infantes, Hipassia M. Moura, Daniele Mantione, Miriam M. Unterlass, David Mecerreyes

**Affiliations:** a POLYMAT, University of the Basque Country UPV/EHU Avenida Tolosa 72 20018 Donostia-San Sebastián Spain david.mecerreyes@ehu.es; b Universität Konstanz, Department of Chemistry, Solid State Chemistry Universitatsstrasse 10 D-78464 Konstanz Germany miriam.unterlass@uni-konstanz.de; c CeMM – Research Center for Molecular Medicine of the Austrian Academy of Sciences Lazarettgasse 14, AKH BT 25.3 1090 Vienna Austria

## Abstract

Energy storage will be a primordial actor of the ecological transition initiated in the energy and transport sectors. As such, innovative approaches to design high-performance electrode materials are crucial for the development of the next generation of batteries. Herein, a novel dual redox-active and porous polyimide network (MTA-MPT), based on mellitic trianhydride (MTA) and 3,7-diamino-*N*-methylphenothiazine (MPT) monomers, is proposed for applications in both high energy density lithium batteries and symmetric all-organic batteries. The MTA-MPT porous polyimide was synthesized using a novel environmentally-friendly hydrothermal polymerization method. Rooted in its dual redox proprieties, the MTA-MPT porous polyimide exhibits a high theoretical capacity making it a very attractive cathode material for high energy density battery applications. The cycling performance of this novel electrode material was assessed in both high energy density lithium batteries and light-weight symmetric all-organic batteries, displaying excellent rate capability and long-term cycling stability.

## Abstract 2

Design of dual redox-active porous polymers towards high-energy density and performance organic electrode materials for lithium metal battery and symmetric all-organic battery applications.

## Graphical abstract

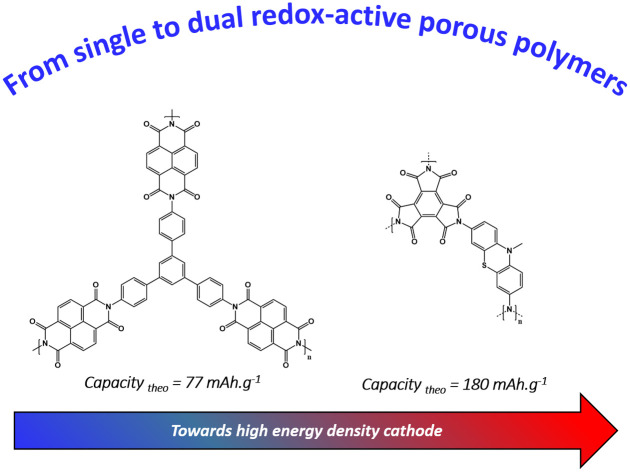

**New concepts**

Herein, a novel synthetic design for organic electrode materials is proposed by introducing for the first time the concept of dual-redox active materials in organic porous materials. This new concept allows the development of organic porous materials with high capacity, corresponding to a 130% increase compared to previous organic porous electrode materials that only exhibits one redox process due to the use of non-redox active A3-cross linker to induce the porosity. This new design concept opens new research direction for the design of practical and high energy density organic porous electrode materials, through the exploration of an infinite combination of redox active A3 cross linker with redox active ligand in order to tune the materials properties to the application requirement (*i.e.* surface area, porosity, pore size, and redox chemistry). We foreseen that this new type of materials could have great potential in applications such as energy storage (Demonstrated in this communication), desalination, water purification and gas separation membrane technologies.

## Introduction

Batteries will play a key role in the technological transitions associated with the energy and transport sectors.^1,2^ As a result, the global battery demand is expected to increase tenfold in the next 10 years,^2^ inducing significant stress on the raw material market for Li-ion batteries (LIBs). In fact, several elements used in LIBs are already critically scarce (*i.e.* lithium, cobalt, nickel, and graphite).^3,4^ Therefore, from an abundance standpoint, organic electrode materials have attracted a lot of attention from the battery community, as they are essentially composed of naturally abundant atoms (*i.e.*, carbon, hydrogen, oxygen, nitrogen and sulphur),^5^ which makes them highly attractive especially compared to the more scarcely available inorganic electrode materials.^6,7^ However, organic electrode materials based on small molecules suffer from high solubility, and hence dissolution into the battery electrolyte, which results in fast capacity fading. One of the most successful approaches to mitigate this issue resides in covalently bonding the redox-active moiety onto a polymeric backbone, reducing its solubility due to the high molecular weight of the polymer chains and/or crosslinking of the polymeric chains.

Over the past few decades, a vast variety of redox polymers has been developed for application in various battery technologies, including alkali metal/ion organic batteries and all-organic batteries.^8–11^ The most studied redox chemistries are based on conjugated carbonyls, conjugated thioethers, conjugated amines, and nitroxide radicals.^5^ Besides the redox chemistry used, the molecular architecture of the redox polymer plays a great role in dictating its electrochemical performance. Recently, a novel subclass of organic electrode materials has emerged based on redox-active porous frameworks, such as porous organic polymers (POPs), conjugated microporous polymers (CMPs) and covalent organic frameworks (COFs), for application in metal-ion batteries (*e.g.*, LIBs) and pseudo-supercapacitors.^12,13^ Redox-active porous networks and frameworks typically exhibit large specific surface area, high physico-chemical stability (incl. insolubility), and permanent pore structure, making them attractive electrode materials for energy storage applications. The high surface area and pore structure in these materials are believed to promote high ionic conductivity within the electrode composite after the liquid electrolyte has filled the networks’ and frameworks’ porosity; reducing diffusion pathways to access the redox active sites. This is not possible in traditional dense redox polymers, where the swellability of the redox polymer is the limiting factor.^12–14^ One of the main drawbacks of redox-active porous networks and frameworks is their relatively low theoretical capacities (≤100 mA h g^−1^) due to the mass of the non-redox-active monomer/ligand required for the formation of a porous network.^12,13^

**Scheme 1:**
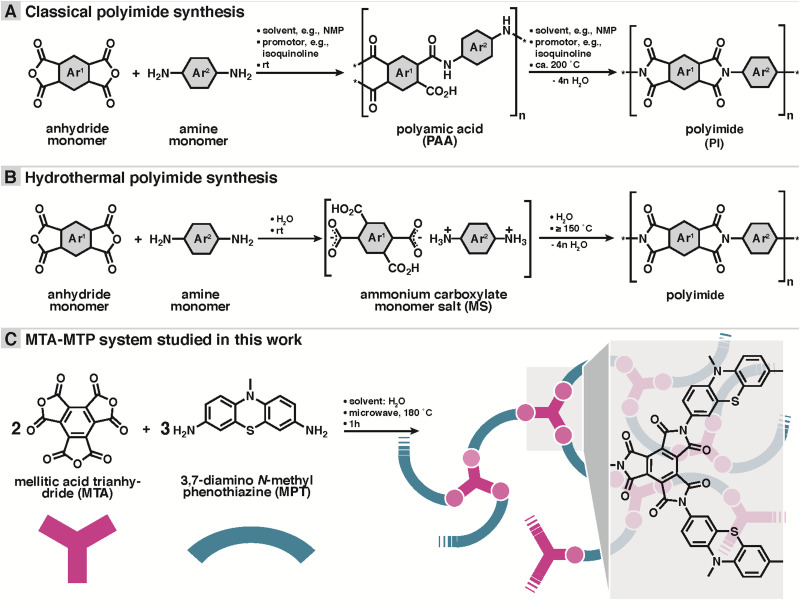
Polyimide syntheses. (A) Classical polyimide (PI) synthesis from anhydride and amine co-monomers *via* polyamic acids (isolable). (B) Hydrothermal PI synthesis from anhydride and amine monomers *via* ammonium carboxylate monomer salts (MS). (B) Typical reaction conditions indicated on arrows. * designates non-specified end-group. (C) Hydrothermal synthesis and designated structure of mellitic trianhydride (MTA) and 3,7-diamino *N*-methyl phenothiazine (MPT)- based PI network systems used in this study. Network schematic: MTA in red, MPT in blue, imide linkages in light red.

Polyimides (PIs) represent one of the most studied redox polymer classes for application in energy storage due to their high physico-chemical stability, high theoretical capacity, good rate capability and long-term cycling stability. PIs’ redox mechanism is based on the enolization of some of the available carbonyl groups, which can be reversibly re-oxidized back to carbonyl moieties. In most cases, PIs are synthesized by cyclocondensation of amine with carboxylic acid dianhydride functional groups, and the used monomers are aromatic/semiaromatic (*cf.*Scheme 1A and B). Tian *et al.* have combined PIs’ redox activity with porosity and reported the synthesis of several porous PIs, based on either 1,2,4,5-benzenete-tracarboxylic anhydride (pyromellitic dianhydride, PMDA), 1,4,5,8-naphthalenete-tracarboxylicdianhydride (NTCDA), or 3,4,9,10-perylene-tetracarboxylicacid-dianhydride (PTCDA), and their application as cathode materials for LIBs.^15^ To induce porosity, they employed the A_3_-type monomer 1,3,5-tris(4-aminophenyl)-benzene (TAPB).^15^ Since the theoretical capacity of an electroactive material (*C*_theo_ [mA h g^−1^]) is inversely proportional to its molecular weight, the use of a non redox-active A_3_-type cross-linker resulted in a significant decrease of *C*_theo_, going from 203 to 77 mA h g^−1^, for the NTCDA monomer and the NTCDA-based porous PI, respectively. Therefore, the benefits of the porosity (*i.e.*, fast ionic diffusion) were in the case of Tian *et al.*'s report undermined by the reduction of the theoretical capacity of the electroactive materials, hampering their application as cathode materials in LIBs.

Herein, a novel dual redox-active porous PI (MTA-MPT) is proposed for application in high-energy density lithium metal batteries. To mitigate the reduction of the theoretical capacity of the resulting porous PI, a redox-active A_3_-type anhydride monomer, mellitic trianhydride (MTA), was used in conjunction with the redox-active B_2_-type amine monomer, 3,7-diamino-*N*-methylphenothiazine (MPT), *cf.*Scheme 1C. As a result, the MTA-MPT porous PI exhibits a theoretical capacity of 180 mA h g^−1^ (based on 4 e^−^), making it a high energy density cathode material. A novel hydrothermal polymerization (HTP) method (*i.e.*, using water as the only solvent in the PI synthesis)^16,17^ was used to generate the MTA-MPT porous PI. HTP provides clear environmental benefits compared to the toxic solvents traditionally used in PI synthesis (*e.g.*, *m*-cresol, dimethylformamide, *N*-methyl-2-pyrrolidone, or dimethylacetamide/toluene).^18–20^ Note that HTP involves different intermediates: while classical PI synthesis involves poly(amic acid) intermediates (*cf.*Scheme 1A), HTP involves isolable monomer salt intermediates (*cf.*Scheme 1B). This difference is simply generated by the polar aprotic solvents in conventional *vs.* protic polar environment in the pre-hydrothermal stages of HTP.

The herein hydrothermally synthesized MTA-MPT porous PI was characterized by means of solid-state NMR spectroscopy, ATR-FTIR spectroscopy, XRD, TGA, BET analysis, and SEM. Additionally, the cycling performance of the MTA-MPT porous PI was assessed in lithium metal battery. Finally, its application and electrochemical performance in lightweight symmetric all-organic battery was also investigated due to its dual-redox properties.

## Results & discussions

The dual redox-active porous PI (MTA-MPT) was synthesized using HTP, as summarized in Scheme 1B and C. The polycondensation reaction between the 3,7-diamino-*N*-methylphenothiazine B_2_-monomer (DA-MPT) and the mellitic trianhydride (MTA) A_3_ monomer was performed in water without any additives at a 180 °C for 1 hour, in a stirred microwave reactor (see ESI† for details). As illustrated in Scheme 1B, HTP is known to proceed *via* isolable ammonium carboxylate salts (aka ‘monomer salts’, MS) as intermediates. MS are typically crystalline and can be prepared on purpose and supplied as precursors. The cation: anion stoichiometry of a MS depends on both the p*K*_a_ difference between NH_2_ and CO_2_H functions and the lattice energy of the salt. However, the cation:anion stoichiometry of a MS is not necessarily identical to the polymerization desired stoichiometry. Therefore, here we chose not to start from a MS, but to supply MTA and DA-MPT directly. A 2 : 3 ratio was chosen, intending to generate a fully imidized, porous network or framework. Note that as aromatic anhydrides are known to typically be existent as an equilibrium mixture of anhydride and free acid, MTA was freshly prepared, thoroughly dried, and stored under Argon prior to the hydrothermal synthesis.

**Fig. 1:**
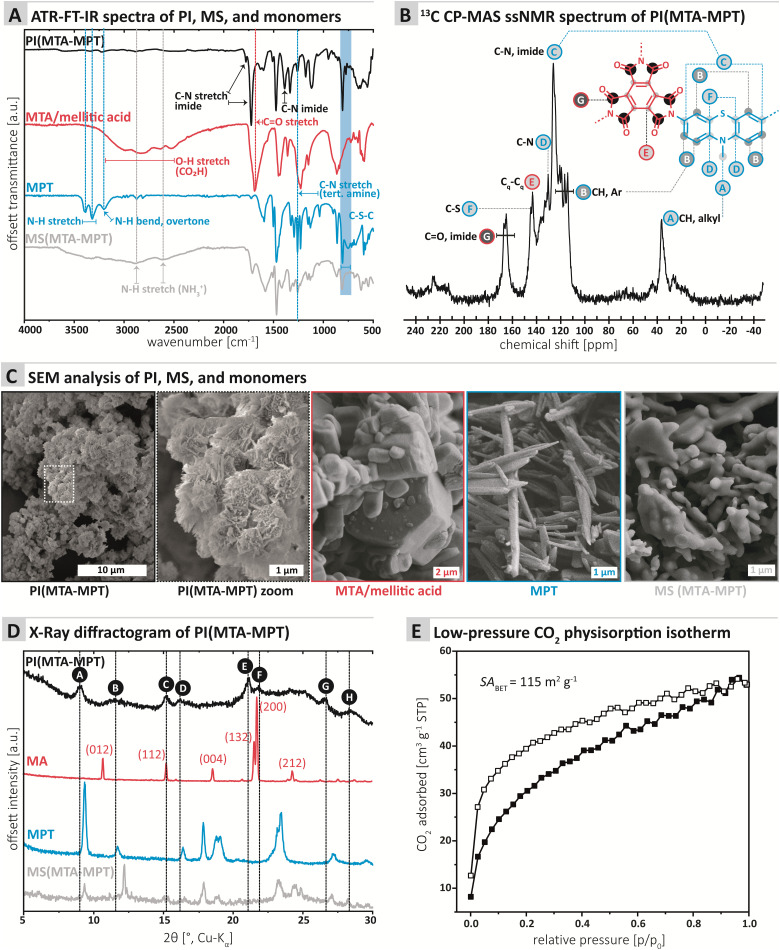
Characterization of the PI MTA-MTP. (A) ATR-FTIR spectra of PI, monomers, and MS, with mode assignment. (B) ^13^C CP-MAS solid-state NMR spectrum of PI MTA-MTP with signal assignment. (C) SEM images of PI, monomers, and MS. (D) Powder X-ray diffractogramm of PI, monomers, and MS. (E) Low-pressure CO_2_ physisorption isotherm (white squares: adsorption; black squares: desorption).

The chemical composition of the PI MTA-MTP was first assessed by ATR-FT-IR (attenuated total reflectance Fourier transform infrared) spectroscopy. Furthermore, we compared PI MTA-MTP's spectrum to those of the unreacted monomers as well as the MS prepared for comparison (see ESI† for MS synthesis). All four spectra are shown in Fig. 1A. The PI (black curve, Fig. 1A) shows the characteristic imide modes, *i.e.*, *ν*(C=O)_as_: 1775, *ν*(C=O)_s_: 1725 cm^−1^, and *ν*(C–N): 1370 cm^−1^. Furthermore, the spectrum shows several very weak modes in the range 3750–2500 cm^−1^, which however do not correspond to amine, CO_2_H, or amide (partial condensation). We hypothesize that these modes correspond to different overtones. Instead of comparing the PI to anhydride's spectrum, we chose to compare to the free acid (mellitic acid, MA), as MTA hydrolyses immediately in water. The MA spectrum (red curve, Fig. 1A) most prominently features several weak modes in the range 3250–2500 cm^−1^, corresponding to hydrogen-bonded carboxylic acid O–H stretches. The spectrum also features a strong but broad carbonyl mode at *ca.* 1700 cm^−1^, which corresponds well to C=O of a carboxylic acid. The MPT monomer's spectrum (blue curve, Fig. 1A) features the two characteristic primary amine N–H stretches at *ca.* 3400 and 3300 cm^−1^, as well as the aromatic primary amines characteristic N–H bending overtone at *ca.* 3250 cm^−1^. A strong mode at *ca.* 1330 cm^−1^ is characteristic for *N*-methyl thiazine's tertiary amine C–N(azine), albeit being a compound mode of C–N, C–H(Ar) and C–H (NH_3_) modes.^21^*N*-Methyl phenothiazine's C–S–C modes are exclusively weak and are present as two modes, of which one strong, in the area of 775–755 cm^−1^, in accordance with the literature.^21^ Note that both the C–N and the C–S–C modes of MPT are nicely retained in both the PI and the MS. The MS (gray curve, Fig. 1A) features the characteristic broad aryl ammonium modes at 2850 and 2580 cm^−1^. Overall, the ATR-FT-IR spectrum of the PI neither features amine nor CO_2_H/anhydride modes, which points at a high degree of condensation. At the same time, both imide and thiazine modes are nicely visible and hence retained in the PI.

**Fig. 2:**
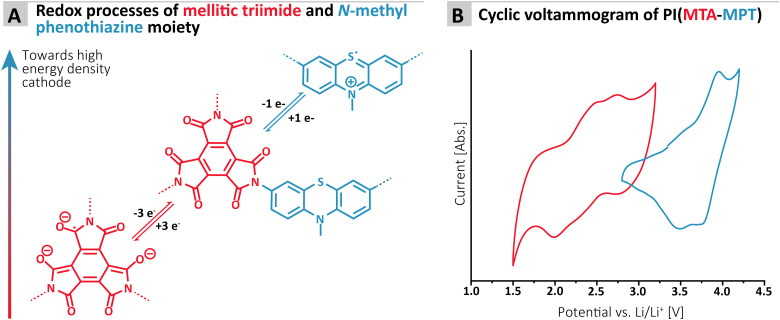
(A) Redox processes associated with the imide and phenothiazine moiety of the MTA-MPT porous polyimide; (B) cyclic voltammogram of the MTA-MPT porous polyimide in lithium-half cell (1 M LiTFSI in DOL/DME as electrolyte; scan rate of 50 mV s^−1^), highlighting its dual redox properties (*i.e.* imide in black and phenothiazine in blue).

The chemical composition of the MTA-MPT PI was further analyzed by ^13^C cross-polarization magic angle spinning (CP-MAS) solid-state NMR spectroscopy (Fig. 1B). The most down-field signal G (167.11–165.13 ppm) is assigned to the quaternary carbonyl carbon.^22^ The signals between 145 and 126 ppm (F, E, D, and C) can be assigned to carbon atoms connected to N (C, D), S (F), and C (E). The aromatic, hydrogen bearing carbons (B) are located between 121 and 114 ppm. Finally, the CH_3_ connected to N within the MPT moiety is found at the most upfield signal A (36.45 ppm).

Furthermore, we performed C,N,H elemental analysis (EA), see ESI.† EA was performed in duplicates of each of two different batches of MTA-MPT porous PI, and C/N ratios of 6.575 (C: 55.79%; N: 9.90%; H: 3.84%) and 6.477 (C: 59.52%, N: 10.72%, H: 3.71%), respectively, were obtained. First, note that the values between the two batches differ significantly, with respect to deviations common for small molecule EA. Second, for a fully imidized PI MTA-MPT network of 2 : 3 stoichiometry a C/N ratio of 7.29 would be expected. At the same time, both ATR-FT-IR and ssNMR spectroscopy do neither point at incomplete condensation (absence of end-groups) nor amide instead of imide linkages. Thus, we suspect that the off ratio of C/N and the variance between samples points at partial and irregular decarboxylation, which would account for the 2 : 3 stoichiometry of monomeric units to remain, whilst the observed C/N ratio would be different from the expected one. To test this hypothesis, we calculated composition and C/N ratios for such scenarios (see ESI†). Indeed, a network of 2 : 3 stoichiometry but with on average one anhydride function per MTA decarboxylating during synthesis would lead to a C/N ratio of 6.56. Consequently, only two out of the three anhydride functions of MTA would have reacted to imides, while the third anhydride function would have decarboxylated. As this is believed to happen on average, the overall network structure would be retained. Neither the ATR-FT-IR nor the ssNMR spectrum of PI MTA-MPT do confirm nor contradict this hypothesis, as decarboxylation would add C_Ar_–H signals, which the PI product would also give without decarboxylation. The decarboxylation scenario aligns with recent reports of decarboxylation of aromatic carboxylic acid species under mild hydrothermal (150–230 °C) conditions,^23^ and has also been put forward recently for polyimide framework syntheses under classical conditions.^24^

Overall, from all chemical analyses, *i.e.*, ATR-FT-IR and ssNMR spectroscopy, and EA, we can at this point only conclude that the product shows dominant imide as well as thiazine functions, which means the redox active functions are intact and no residue of intermediate monomer salts are present. Rather, we hypothesize, but cannot claim from the data with certainty, that some degree of inhomogeneous decarboxylation occurs during PI MTA-MPT's synthesis, which would lead to organic Bertholide-like compounds. Therefore, we expect the PI MTA-MPT to act as an ionic and electronic insulator with redox proprieties.

**Fig. 3:**
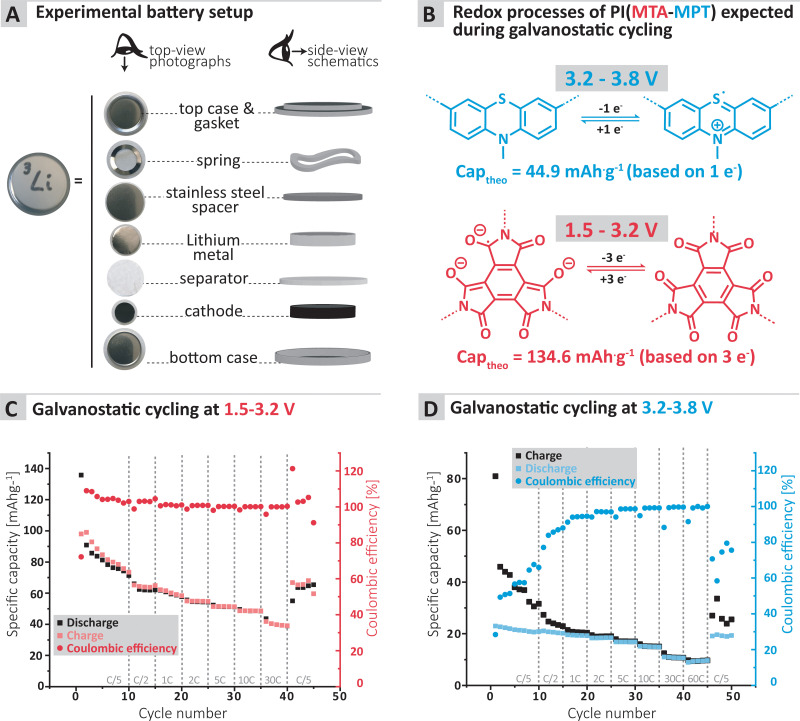
(A) Experimental setup used to assess the cycling performance of the MTA-MPT porous polyimide as well as (B) the redox mechanism expected to occur for each redox processes during cycling. Galvanostatic cyling of the MTA-MPT porous polyimide in a lithium metal battery at various C-rate with different voltage ranges: (C) 1.5 V–3.2 V (imide process), (D) 3.2–3.8 V (phenothiazine process).

Next, we assessed the morphology and crystallinity of the MTA-MPT PI by scanning electron microscopy (SEM) and powder X-ray diffraction (PXRD), and compared these to the pristine monomers and the MS prepared for comparison (*cf.*Fig. 3C and D, respectively). SEM evinced that all morphologies were highly homogenous. The PI is composed of microparticles of *ca.* 1 μm in diameter, decorated with nanoscopic angular particles (Fig. 1C). The morphologies (Fig. 1C) of both monomers and MS are entirely different: MTA is composed of hexagonal prismatic crystallites of isometric habit with sizes ranging from *ca.* 1 to 5 μm. MPT is composed of needle structures typical for aromatic amines (*ca.* 100 nm in diameter, several micron in length). The MS in contrast consists of partly coalesced aggregates (several micron in size) of roundish particles of *ca.* 1–2 μm diameter. For the samples individual morphological homogeneity but strong morphological difference between PI, monomers, and MS, we conclude that the PI does not contain significant amounts of either of the starting compounds/intermediates. Furthermore, the PI micromorphology points towards some degree of crystallinity (angularity of the features).

PXRD data (Fig. 1D) shows a surprising result that is however in line with the SEM data: Several reflections, albeit broad and weak and accompanied by an amorphous background, are present. Reflections A, C, E, F, and G (Fig. 1D) are comparably narrow, whilst peaks B, D, and H, are rather broad. Clearly, some, albeit low, degree of order is present in PI MTA-MPT. Comparison with the diffractograms of MA (red. Fig. 1D; indexed according to ref. 25 CCDC Refcode: 1210972), MPT (blue, Fig. 1D) and the MS (gray, Fig. 1D) shows that only one reflection in the PI product's diffractogram coincides with reflections of the precursors: Reflection C at *ca.* 15°(2*θ*, Cu-Kα) coincides with a reflection in MA (red curve). In MA's diffractogram, the reflection corresponds to (112),^25^ which is significantly less intense than the major reflection in MA's pattern, *i.e.* (002), at *ca.* 21°(2*θ*, Cu-Kα). Consequently, if the reflection C in the PI product would belong to unreacted MA, MA's (002) reflection would also have to be present. This is not the case and hence we conclude that C only accidentally coincides with (112) of MA. No other reflections of MS or pristine monomers coincide with the PI product's diffractogram. Hence, the reflections and peaks of PI(MTA-MPT) arise from the material itself and not from crystalline starting compound/intermediate compounds. Note that the PI's order is quite surprising, for the potential partial decarboxylation. Note that to date, only two reports of the HTS of imide-linked network structure exist, one by our own group generating amorphous networks under any tested conditions,^16^ and a recent report by Kim and coworkers, reporting the HTS of imide-linked COFs, *i.e.*, highly crystalline frameworks.^26^ In the present case, we believe the structure to be consisting of ordered framework domains (minority) coexisting with amorphous network domains (majority). The synthesized PI is not a COF as it lacks the necessary reflections at lower angles (not depicted in Fig. 1D).

Next, gas sorption experiments were performed (Fig. 1E and ESI† for details). A porous structure can be hoped for when employing a stiff A_3_-type monomer like MTA. On the other hand, both COFs and especially amorphous polymer networks often have interpenetrated structures,^27^ which decreases the overall porosity. The Brunauer–Emmett–Teller surface area (SA_BET_) of PI (MTA-MPT) was determined to be 115 m^2^ g^−1^. In comparison, Tian *et al.*^15^ obtained SA_BET_ of 508 and 200 m^2^ g^−1^ for two of their porous PI networks, and the absence of porosity for the third system.^15^ The pore size distribution (PSD; Monte-Carlo Method at 273 K) points at two pore populations around 0.5 nm and 0.8 nm (see ESI† for PSD plot). For a fully condensed crystalline PI network, we would expect a pore size of *ca.* 1.4 nm. Both 0.5 nm and 0.8 nm would correspond well to an interpenetrated network (see ESI† for calculation). Overall, it can be said that PI MTA-MPT is porous, which we deemed highly promising in combination with the potential dual redox-activity provided by the imide and the thiazine functions.

As final materials characterization, we assessed the thermal stability of PI (MTA-MPT) by thermogravimetric analysis (TGA). The PI exhibits a 10% weight loss at 273 °C (T90%), which is significantly lower than typical for conventional aromatic PIs (*i.e.*, T90% ≥ 450 °C). We believe that this is a direct consequence of the presence of the phenothiazine moiety, which is expected to show – in comparison to fully aromatic high-performance polymers – rather low thermal stabilities. Nevertheless, the MTA-MPT polyimide displays sufficient thermal stability for battery operation, where temperatures never exceed 100 °C.

To assess the redox proprieties of the MTA-MPT porous PI, cyclic voltammetry (CV) was performed using a 1 M lithium bis(trifluoro-methanesulfonyl)imide (LiTFSI) in 1,3-dioxolane (DOL)/1,2-dimethoxyethane (DME), as electrolyte in a lithium coin cell. The results are shown in Fig. 2. As expected, the porous PI MTA-MPT exhibits two redox processes located around 2.25 V and 3.70 V *vs.* Li/Li^+^, respectively. The former corresponds to the reversible enolization process of carbonyl groups present in the MTA moiety, while the latter is related to the reversible formation of a radical cation of the MPT moiety, as illustrated in Fig. 2A. As a result of the dual redox proprieties of MTA-MPT porous PI, a theoretical capacity of 180 mA h g^−1^ is expected (based on 4 e^−^: 3 e^−^ and 1 e^−^ for the imide and phenothiazine processes, respectively), making it a very promising high energy density cathode material.

To further confirm the applicability of the MTA-MPT porous PI as cathode material in lithium metal batteries, galvanostatic cycling of a MTA-MPT porous PI-based electrode was performed as shown in Fig. 3. First, the cycling performance of the imide and phenothiazine processes were assessed separately by using different voltage cut-off ranges, of 1.5–3.2 V and 3.2–3.8 V *vs.* Li/Li^+^, respectively (Fig. 3C and D). As can be seen in Fig. 3, the imide redox process of the MTA-MPT porous PI delivers an initial specific discharge capacity of 136 mA h g^−1^ at a C-rate of C/5, which corresponds to the theoretical capacity of the imide redox process of MTA-MPT porous PI. (Note that with respect to our earlier discussion of the MTA-MPT composition and stoichiometry, this is a strong indicator for the desired 2 : 3 stoichiometry and major imidization *vs.* only some minor amide function presence). However, a capacity fading is observed upon cycling, reaching a specific discharge capacity of 71 mA h g^−1^ after 10 cycles. Upon increasing C-rate from C/2 to 30 C, the MTA-MPT polymer displays stable specific discharge capacities of 62, 58, 54, 51, 48 and 39 mA h g^−1^ at C-rates of C/2, 1C, 2C, 5C, 10C and 30C, respectively, highlighting the high rate capability of the imide redox process. Interestingly, a stable specific discharge capacity of 65 mA h g^−1^ is observed when cycling back the MTA-MPT polymer at a C-rate of C/5, corresponding to a capacity retention of 91.7%. This slight reduction in capacity suggests that very minor deterioration of the electrode microstructure or degradation of the MTA-MPT PI has occurred when cycled at high C-rate. For the phenothiazine redox process, very stable specific discharge capacities of 21, 20, 19, 18, 17, 15, 11 and 10 mA h g^−1^ are observed at C-rates of C/5, C/2, 1C, 2C, 5C, 10C, 30C and 60C, respectively. Compared to the imide redox process, the phenothiazine one exhibits a moderate coulombic efficiency (CE, *i.e.* 75–85%) at low C-rate (≤C/2), which improves upon increasing C-rate, reaching CE values of 94% and 99% at a C-rate of 1C and 60 C, respectively. It is unclear to this stage, if the low CE obtained at low C-rate is due to inherent proprieties of the phenothiazine redox moity or due to the low voltage cut-off used to assess only the phenothiazine redox process (*i.e.* not sufficient voltage separation between the imide and phenothiazine processes to study them separately). The latter is more likely, based on the CE close to 100% obtained when cycling both redox processes together as discussed in following section.

**Fig. 4:**
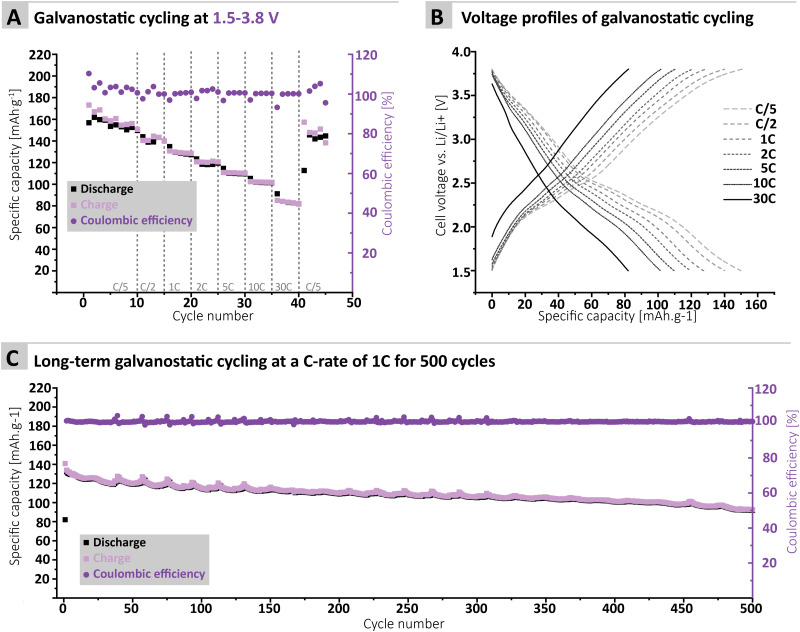
(A) Galvanostatic cyling of the MTA-MPT porous polyimide in a lithium metal battery at various C-rate with a voltage ranges of 1.5 V–3.8 V (both imide & phenothiazine processes) and (B) its corresponding voltage profile. (C) Long-term cycling at a C-rate of 1C for 500 cycles.

Furthermore, the MTA-MPT electrode was cycled over a voltage range covering both the imide and the phenothiazine redox processes (*i.e.*, 1.5–3.8 V *vs.* Li/Li^+^), see Fig. 4. Interestingly, a relatively stable specific discharge capacity of around 155 mA h g^−1^ is observed in this case, with a CE close to 100%. This contrasts with the capacity fading observed when cycling the MTA-MPT polymer with a limited voltage range (*i.e.* 1.5–3.8 V *vs.* Li/Li^+^) in order to assess only the imide redox contribution. Upon increasing C-rate, the MTA-MPT polymer displays high rate capability, delivering 53% of its initial capacity at a C-rate of 30C when compared to a C-rate of C/5. When cycling back to a C-rate of C/5, a capacity retention of 96.6% is observed, suggesting that very little to no deterioration of the electrode microstructure or degradation of the MTA-MPT PI has occurred when cycled at high C-rate. Additionally, a CE close to 100% is reached over the whole range of C-rate, ranging from C/5 to 30C, highlighting the high reversibility of both redox processes. The MTA-MPT porous PI also displays good cycling stability, delivering an initial specific discharge capacity of 132 mA h g^−1^, of which 70% remains after 500 cycles at a C-rate of 1C (see Fig. 4C). For comparison purposes, the cycling performance of MTA-MPT porous PI was also assessed using a more traditional carbonate electrolyte (*i.e.* 1 M lithium hexafluorophosphate (LiPF_6_) in ethylene carbonate (EC)/diethyl carbonate (DEC)), generally used in lithium-ion batteries. (see ESI† for details) Overall, slightly lower specific discharge capacities are obtained with the carbonate-based electrolyte for all c-rate studied, when compared to the results obtained with ether-based electrolyte (*e.g.* 155 *vs.* 142 mA h g^−1^ for 1 M LiTFSI DOL/DME and 1 M LiPF_6_ EC/DEC, respectively) Additionally, slightly lower rate capability is also observed with the carbonate-based electrolyte, retaining 42% of its initial capacity at a C-rate of 30C when compared to a C-rate of C/5. Such results are in agreement with our previous work on redox polymer based on polyimide.^19^ Overall, the MTA-MPT polymer shows excellent performance in the lithium metal battery, highlighting the great potential of this porous polyimide as high energy density cathode material.

**Fig. 5:**
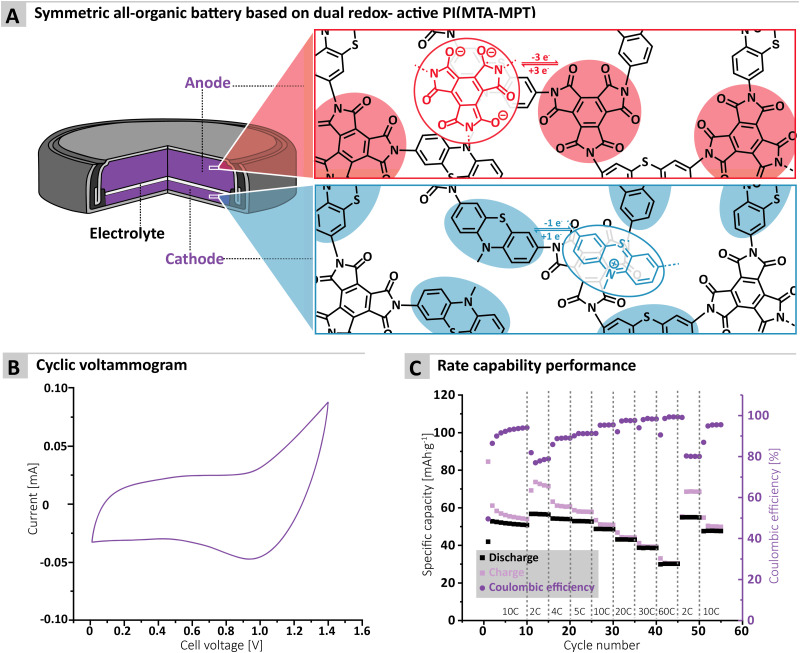
(A) Scheme illustrating the principle of a symmetric all-organic battery, based on the MTA-MPT porous polymer, (B) cyclic voltammetry of the resulting symmetric all-organic cell as well as (C) its rate capability performance.

Finally, the cycling performance of the MTA-MPT porous PI in a symmetric all-organic battery was assessed, as its dual redox properties should allow for its use as both anode and cathode materials. Fig. 5A shows the working principle of a symmetric all-organic battery based on the MTA-MPT porous PI, where the imide redox process is employed in the anode, while the phenothiazine redox process is used by the cathode. As can be seen in Fig. 4B, the voltage range used for cycling this symmetric cell was determined by cyclic voltammetry, resulting in a 1.4 V range, which is in accordance with potential difference between the imide and phenothiazine redox processes. Fig. 4C shows the galvanostatic cycling performance of the symmetric cell, which is anode limited by design (*i.e.* Cap_theo_ = 134.6 mA h g^−1^), at various C-rates. The symmetric cell delivered an initial specific discharge capacity of 53 mA h g^−1^ at a C-rate of 10C, which remains stable up 10 cycles. Despite a low CE for the first cycle (*i.e.*, 50%), CE significantly improves upon cycling, reaching a CE value of 94% after 10 cycles. Decreasing the C-rate to 2C results in a small increase of the specific discharge capacity to 57 mA h g^−1^, although this coincides with a decrease of the CE to 79%. Upon increasing the C-rate from 2C to 60C, the symmetric cell delivers stable specific discharge capacities and displays high rate performance with a capacity retention of 54% at 60C when compared to 2C. Despite the symmetric all-organic battery based on the MTA-MPT porous PI displaying moderate specific discharge when compared to its theoretical capacity (*i.e.* 42% at a C-rate of 10C), the symmetric cell shows good cycling stability and high rate performance. Therefore, such symmetric all-organic battery could be very attractive for light-weight energy storage applications.

## Conclusions

Herein, a dual redox-active porous polyimide has been synthesized using an environmentally-friendly hydrothermal polymerization method for applications in high energy density lithium metal batteries and symmetric all-organic batteries. To induce porosity, a novel redox-active A_3_-type cross-linker based on mellitic trianhydride was employed in conjunction with a redox-active ligand based on 3,7-diamino-*N*-methylphenothiazine. The MTA-MPT PI displays good agreement with 2 : 3 stoichiometry, and potentially a small degree of decarboxylation and/or amide functions. Structurally and morphologically, we find, surprisingly, some degree of order, and homogenous flower-like micromorphologies, respectively. The MTA-MPT porous polyimide displays good thermal stability, albeit low surface area that is however similar to previously reported SA_Bet_ of structurally comparable yet single redox-active polyimides.^15^ Additionally, the MTA-MPT porous polyimide exhibits high theoretical capacity, corresponding to a 133% increase compared to previously reported porous PI networks, making it a very attractive cathode material for high energy density batteries. A lithium metal battery based on the MTA-MPT porous PI cathode was constructed and its cycling performance assessed. The MTA-MPT-based cathode exhibits a high and stable specific discharge capacity of 155 mA h g^−1^ at a C-rate of C/5 with a coulombic effiency close to 100%. Additionally, the lihitum metal battery based on MTA-MPT PI cathode displays high rate capability, with a 53% capacity retention even at an elevated C-rate of 30 C. Moreover, the MTA-MPT porous PI cathode shows excellent cycling stability, retaining 70% of its initial capacity upon 500 cycles at a C-rate of 1C. Finally, we demonstrated the applicability of the MTA-MPT-based electrode material in symmetric all-organic batteries, which are an attractive battery technology for lightweight energy storage applications. The symmetric battery, based on the MTA-MPT polymer, displays a cell voltage of 1.4 V, which is in accordance with the standard redox potential of both imide and phenothiazine redox processes. The symmetric cell displays a moderate specific discharge capacity of 57 mA h g^−1^ at a C-rate of 2C, corresponding to 42% of its theoretical capacity (*i.e.*, anode limited by design, referring to the imide redox process). A capacity retention of 54% is observed at a very high C-rate of 60C, highlighting the excellent rate capability of this symmetric battery. Overall, the MTA-MPT porous polyimide displays excellent cycling performance in both high energy density lithium metal battery and symmetric all-organic battery, making it very attractive electrode materials for the next generation of batteries.

## Author contributions

N. Goujon, M. M. Unterlass and D. Mecerreyes designed this project. D. Mantione synthesized and characterized the MPT monomer, and N. Goujon performed all electrochemical characterizations and battery testing. N. Goujon analysed all electrochemical and battery data. M. Lahnsteiner performed hydrothermal synthesis, ATR-FT-IR and PXRD analysis of the PI. H. M. Moura performed SEM, ssNMR, and EA characterization of the PI. D. A. Cerrón-Infantes synthesized the MS and performed SEM, ATR-FTIR, and PXRD analysis of the pristine monomers and the MS. M. Lahnsteiner, D. A. Cerrón-Infantes, H. M. Moura, and M. M. Unterlass analysed all chemical and structural characterization data of the PI. N. Goujon and D. Mecerreyes wrote the first draft of the manuscript. M. M. Unterlass prepared all figures. All co-authors contributed to writing the manuscript.

## Conflicts of interest

There are no conflicts to declare.

## Supplementary Material

MH-010-D2MH01335E-s001

## References

[cit1] I. E. A. (IEA) , World Energy Outlook 2020, 2020

[cit2] Zhao Y., Pohl O., Bhatt A. I., Collis G. E., Mahon P. J., Rüther T., Hollenkamp A. F. (2021). Sustainable Chem..

[cit3] European Commission , Critical Raw Materials Resilience: Charting a Path towards greater Security and Sustainability, https://eur-lex.europa.eu/legal-content/EN/TXT/?uri=CELEX:52020DC0474

[cit4] Giovanni B. , Darina B., Jo D., Cristina T. D. M., Viorel N., Beatriz V. L., Cynthia L., Yildirim K., Laura T. P., Claudia B., Simone M., Lucia M., Philip N., Alain M., Patricia A. D., Claudiu P., Evangelos T., Fabrice M., David P. and Constantin C., European Commission, Assessment of the methodology for establishing the EU list of critical raw materials, https://op.europa.eu/s/oAOn

[cit5] Poizot P., Gaubicher J., Renault S., Dubois L., Liang Y., Yao Y. (2020). Chem. Rev..

[cit6] Guyomard D., Tarascon J.-M. (1994). Adv. Mater..

[cit7] Zhang H., Li C., Eshetu G. G., Laruelle S., Grugeon S., Zaghib K., Julien C., Mauger A., Guyomard D., Rojo T., Gisbert-Trejo N., Passerini S., Huang X., Zhou Z., Johansson P., Forsyth M. (2020). Angew. Chem., Int. Ed..

[cit8] Häupler B., Wild A., Schubert U. S. (2015). Adv. Energy Mater..

[cit9] Muench S., Wild A., Friebe C., Häupler B., Janoschka T., Schubert U. S. (2016). Chem. Rev..

[cit10] Poizot P., Dolhem F., Gaubicher J. (2018). Curr. Opin. Electrochem..

[cit11] Goujon N., Casado N., Patil N., Marcilla R., Mecerreyes D. (2021). Prog. Polym. Sci..

[cit12] Zhang Y., Riduan S. N., Wang J. (2017). Chem. – Eur. J..

[cit13] Liu X., Liu C. F., Lai W. Y., Huang W. (2020). Adv. Mater. Technol..

[cit14] Molina A., Patil N., Ventosa E., Liras M., Palma J., Marcilla R. (2020). Adv. Funct. Mater..

[cit15] Tian D., Zhang H.-Z., Zhang D.-S., Chang Z., Han J., Gao X.-P., Bu X.-H. (2014). RSC Adv..

[cit16] Lahnsteiner M., Caldera M., Moura H. M., Cerrón-Infantes D. A., Roeser J., Konegger T., Thomas A., Menche J., Unterlass M. M. (2021). J. Mater. Chem. A.

[cit17] Baumgartner B., Bojdys M. J., Unterlass M. M. (2014). Polym. Chem..

[cit18] Song Z., Zhan H., Zhou Y. (2010). Angew. Chem., Int. Ed..

[cit19] Hernández G., Salsamendi M., Morozova S. M., Lozinskaya E. I., Devaraj S., Vygodskii Y. S., Shaplov A. S., Mecerreyes D. (2018). J. Polym. Sci., Part A: Polym. Chem..

[cit20] Casado N., Mantione D., Shanmukaraj D., Mecerreyes D. (2019). ChemSusChem.

[cit21] Alcolea Palafox M., Gil M., Nez J. L., Tardajos G. (2002). Int. J. Quantum Chem..

[cit22] Martínez-Richa A., Vera-Graziano R. (1998). J. Appl. Polym. Sci..

[cit23] Amaya-García F., Unterlass M. M. (2022). Synthesis.

[cit24] Kuehl V. A., Wenzel M. J., Parkinson B. A., de Sousa Oliveira L., Hoberg J. O. (2021). J. Mater. Chem. A.

[cit25] Darlow S. F. (1961). Acta Crystallogr..

[cit26] Kim T., Joo S. H., Gong J., Choi S., Min J. H., Kim Y., Lee G., Lee E., Park S., Kwak S. K., Lee H., Kim B. (2022). Angew. Chem., Int. Ed..

[cit27] Ma T., Li J., Niu J., Zhang L., Etman A. S., Lin C., Shi D., Chen P., Li L. H., Du X., Sun J., Wang W. (2018). J. Am. Chem. Soc..

