# Supplementary material for: Dual redox-active porous polyimides as high performance and versatile electrode material for next-generation batteries
Source: Mater Horiz. 2023 Jan 9;10(3):967–76. doi: 10.1039/d2mh01335e (PMC9986975; doi:10.1039/d2mh01335e)
Supplement: MH-010-D2MH01335E-s001 [file MH-010-D2MH01335E-s001.pdf]

Electronic Supplementary Material (ESI) for Materials Horizons.
This journal is © The Royal Society of Chemistry 2023

Supporting information

Dual redox-active porous polyimide for application in lithium metal batteries and symmetric all-organic batteries

Nicolas Goujon,[a] Marianne Lahnsteiner,[b,c] Daniel A. Cerrón-Infantes,[b,c] Hipassia M. Moura,[b,c] Daniele Mantione,[a] Miriam M. Unterlass *[b,c] and David Mecerreyes *[a]

a. POLYMAT, University of the Basque Country UPV/EHU, Avenida Tolosa 72, 20018 Donostia-San Sebastián, Spain. E-mail: david.mecerreyes@ehu.es

b. Universität Konstanz, Department of Chemistry, Solid State Chemistry, Universitaetsstrasse 10, D-78464 Konstanz, Germany. E-mail: miriam.unterlass@uni-konstanz.de

c. CeMM – Research Center for Molecular Medicine of the Austrian Academy of Sciences, Lazarettgasse 14, AKH BT 25.3, 1090 Vienna, Austria.

Table of content

Experimental section 2
Materials 2
Methods 2
Monomer synthesis 4
Monomer salt synthesis 9
Synthesis of dual redox-active porous polyimide 10
Result and discussions 11
Elemental analysis 11
Pore size distribution and hypothesis for pore sizes 13
SEM images 15
lithium metal │MTA-MPT porous PI battery cycling 22
References 23

Experimental section

Materials

Phenothiazine, anhydrous 1,2-dimethoxyethane (DME; 99.5 %) and 1,3-dioxolane (DOL; 99.8 %) was purchased from Sigma Aldrich. Tetrahydrofuran, Mellitic acid and acetic anhydride were purchased from TCI. Lithium bis(trifluoromethanesulfonyl)imide (LiTFSI) was purchased from Iolitec. Battery electrolyte (1 M lithium hexafluorophophate in ethylene carbonate/diethyl carbonate, 50/50 vol.) was purchased from Solvionic. Carbon coated aluminium foil (width 260 mm, thickness 20 µm), lithium metal (width 60 mm, thickness 100 µm), polyvinylidene difluoride (PVDF; Solef® 5130) and conductive carbon black super C65 (Timical) was purchased from Gelon energy Co. Limited. All chemicals and solvents were used as received, without further purification.

Methods

IR spectroscopy measurements were recorded with a Bruker Tensor 27working in ATR MicroFocusing MVP-QL with a diamond crystal. Resolution was set to 4 cm^{-1} and spectra were recorded from 4000 cm^{-1} to 600 cm^{-1}. The data was processed with Spectragryph optical spectroscopy software.

Thermogravimetric analysis was carried out using a Netzsch TG 209 analyzer at a heating rate of 10 K min^{-1} under nitrogen atmosphere, equipped with NETZSCH Proteus (Version 4.3) software.

Powder X-Ray diffraction was performed using a PANalyticalX'Pert Pro multi-purpose diffractometer (MPD) in Bragg Brentano geometry operating with a Cu anode at 45 kV, 40 mA and an X-Celerator multichannel detector. Samples were ground and mounted as loose powder on silicon single crystal sample holders. The diffraction patterns were recorded between 1 and 30° (2θ) with 74.970 s/step and a step size of 0.0201°. The sample holders were rotated during the measurement with 4 s/turn. Powder X-ray diffraction (pXRD) measurements of the powders were also carried out in a Bruker D8 Discover, with a IµS microfocus X-ray source (Cu-Kα radiation) at 50 kV and 100 mA, equipped with energy dispersive LynxEye detector. The samples were measured with 2s per steps, and step size 0,02°, and the samples oscillated in a range of 0,15mm in x and y (not rotated).

Scanning electron microscopy images were acquired in a field emission scanning electron microscope (SEM) Analytic SEM Zeiss Gemini 500 microscope, using 5kV acceleration. Prior to the analysis, the powders were sprinkled over a carbon tape, and the samples were coated with a 17 nm layer of Au/Pd 60/40 alloy in a Safematic CCU-010 LV Low Vacuum sputtering device.

Gas sorption Low pressure CO_2 physisorption isotherms were measured volumetrically at 195 K and 273 K up to 1 bar using an Autosorb-IQ-MP from Quantachrome equipped with a Quantachrome CryoCooler for temperature regulation. Isotherm points chosen to calculate the BET surface area were subject to the consistency criteria detailed by Rouquerol.[1] The pore size distribution was derived from the adsorption isotherms at 273 K using the Monte Carlo model for carbon adsorbents.

^{13}C Solid-state NMR analysis were performed on a Bruker AVANCE 400 (100.6 MHz) spectrometer equipped with a 4 mm broadband MAS probe head and with a spinning frequency of 10 kHz. Spectra were recorded using CP-MAS (cross polarization magic angle spinning) experiment. The spectrum was recorded with 30720 scans, using relaxation delay of 5 s, and contact time of 2000 μs.

Elemental analysis (C, H, and N contents) of two different batches of materials were carried out in a Unicube® organic elemental analyzer. Therefore, ca. 0.1 mg of sample were weighted in tin boats.

Electrode formulation The MTA-MPT-based electrodes were prepared using a doctor blade coating method onto a carbon coated aluminium current collector (Gelon energy Co. Limited, 11 μm thick). For this purpose, a slurry of MTA-MPT porous polyimide (40 wt.%), conductive carbon (Super C65, Timical, 40 wt.%) and binder (PVDF, Solef® 5130, 20 wt.%) in N-methylpyrrolidone (NMP) was prepared using a speed mixer at a rpm of 3000 for 5 mins after each solvent addition (SpeedMixer™, DAC 300-100-SE). Prior solvent mixing, all dried components of the slurry were mixed with a ball mill machined at a rpm of 500 for 5 mins (FRITSCH, Planetary Micro Mill PULVERISETTE 7). MTA-MPT-based electrodes were dried under high vacuum at 50 °C for 24h, resulting in an active material loading around 1.8 $mg.cm^{-2}$.

Electrochemical characterization: Lithium metal batteries, based on MTA-MPT cathode, were assembled inside an argon glovebox using a lithium electrode (Gelon

energy Co. Limited, 11 μm thick) and a cathode electrode of a diameter of 11 mm. Glass microfiber filter (Whatman™, GF/B, 16 mm in diameter) soaked in either 1 M LiTFSI in DOL/DME (50/50 vol%) or 1 M $LiPF_6$ in EC/DEC (50/50 vol%) was used as separator. Cyclic voltammetry (CV) and galvanostatic measurements were performed in a lithium metal coin cell using a multi-channel Potentiostat (Biologic, VMP3) or an battery cycler (Neware), respectively. For the symmetric all-organic batteries, the MTA-MPT-based electrodes were both used as anode and cathode and Glass microfiber filter soaked in 1 M LiTFSI in DOL/DME (50/50 vol%) was used as separator.

Monomer synthesis

10-methyl-10H-phenothiazine-3,7-diamine (DA-MPT):

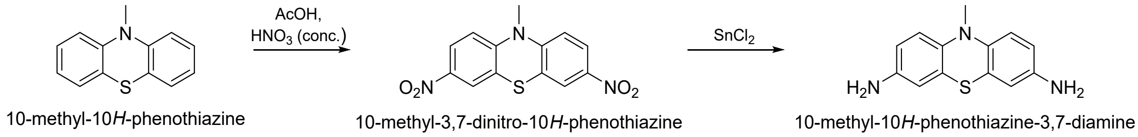

Synthesis of 10-methyl-3,7-dinitro-10H-phenothiazine[2]

The synthesis of 10-methyl-3,7-dinitro-10H-phenothiazine has been performed as previously describe in literature with some facilitations: in a 100mL round bottom flask, 10g (0.0469 mol, 1eq) of 10-methyl-10H-phenothiazine was suspended in 50mL of glacial acetic acid. Then, an excess of nitric acid 67%, 25mL, was added dropwise with a pressure-compensating dropping funnel. To avoid overheating, a water/ice bath has been placed underneath only during the dropping. The mixture become clear and after some minutes a precipitate start to form; the obtained suspension has been let at room temperature stirring for 48 hours. The obtained yellow greenish mixture has been pure into 500mL of water and basified with a saturated solution of sodium carbonate, until basic pH. The resulting solid has been filtered with a Buckner funnel and wash abundantly with water and isopropanol. The resulting yellow solid has been dried in vacuum, to yield 11,62g (82%) of bright yellow fine powder. 1H NMR (300 MHz, DMSO) δ 8.97 (d, J = 2.7 Hz, 2H), 8.56 (dd, J = 9.3, 2.7 Hz, 2H), 7.94 (d, J = 9.4 Hz, 2H), 3.98 (s, 3H). 13C NMR (75 MHz, DMSO) δ 142.49, 141.92, 127.94, 126.80, 124.56, 118.44, 36.95.

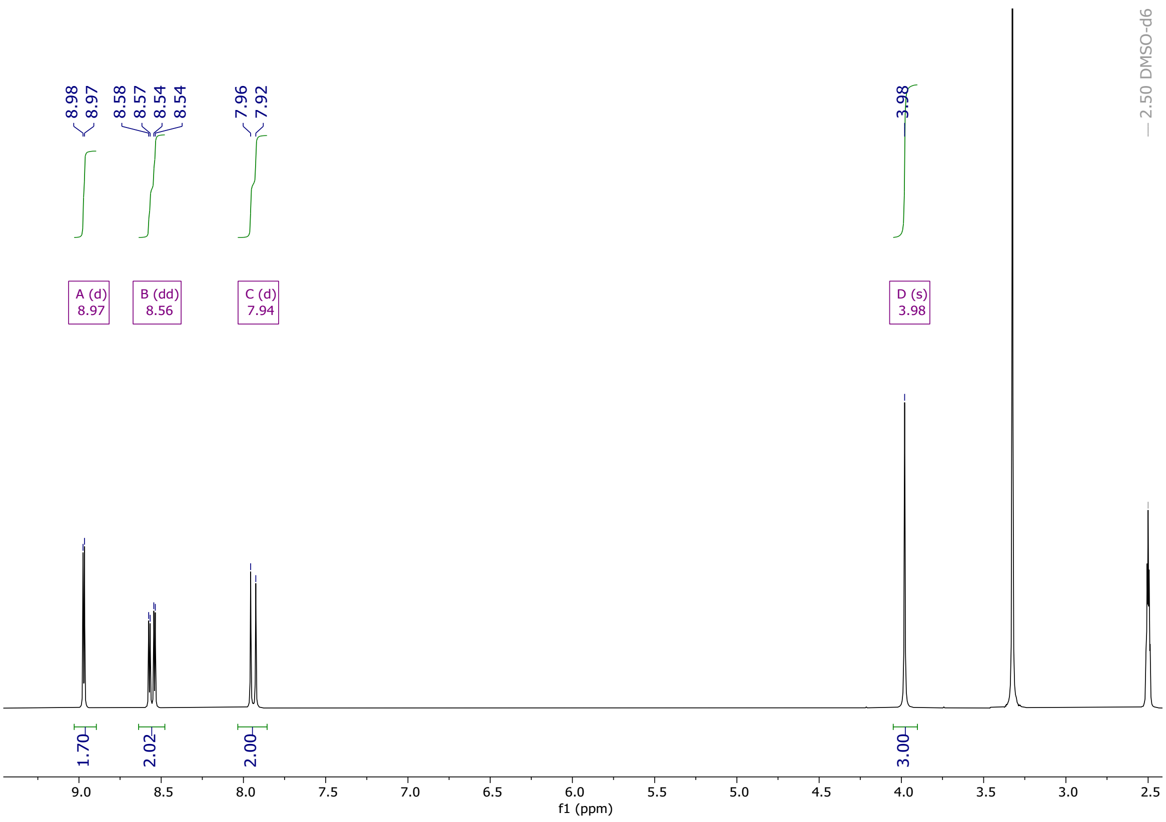

Figure S 1. ^{1}H NMR of 10-methyl-3,7-dinitro-10H-phenothiazine

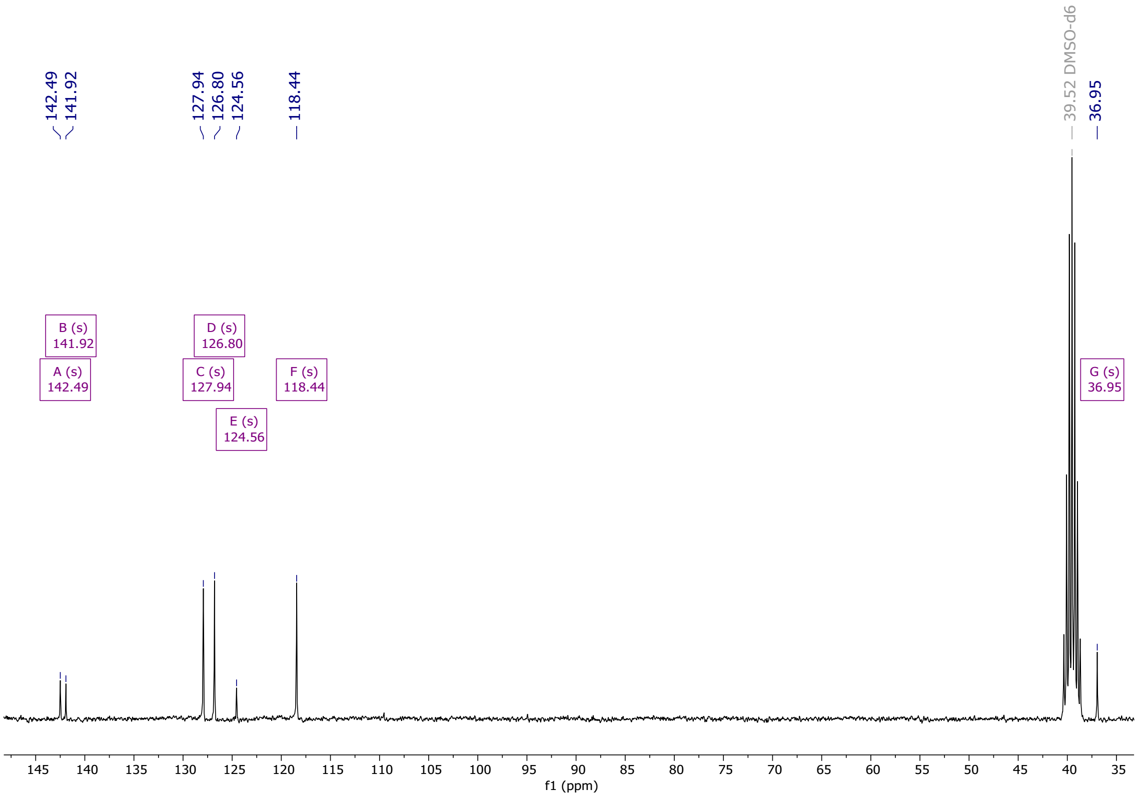

Figure S 2. ^{13}C NMR of 10-methyl-3,7-dinitro-10H-phenothiazine

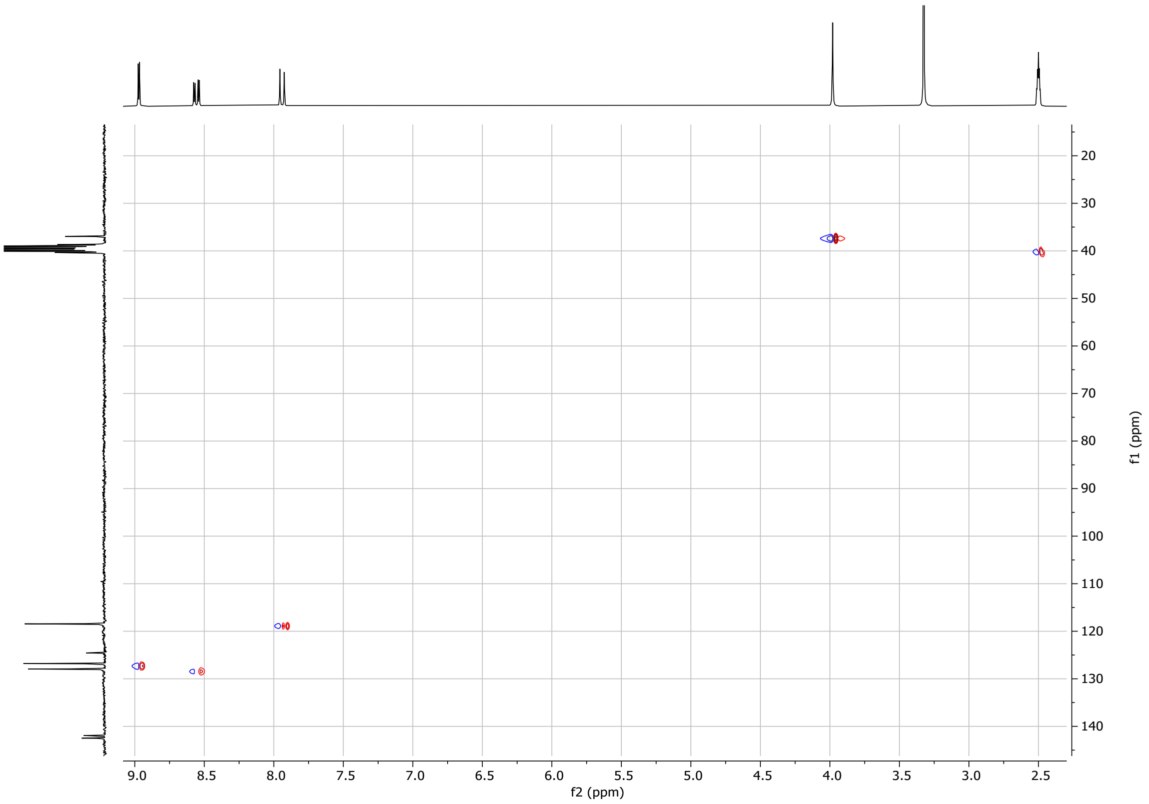

Figure S 3. ^{1}H-^{13}C HSQC of 10-methyl-3,7-dinitro-10H-phenothiazine

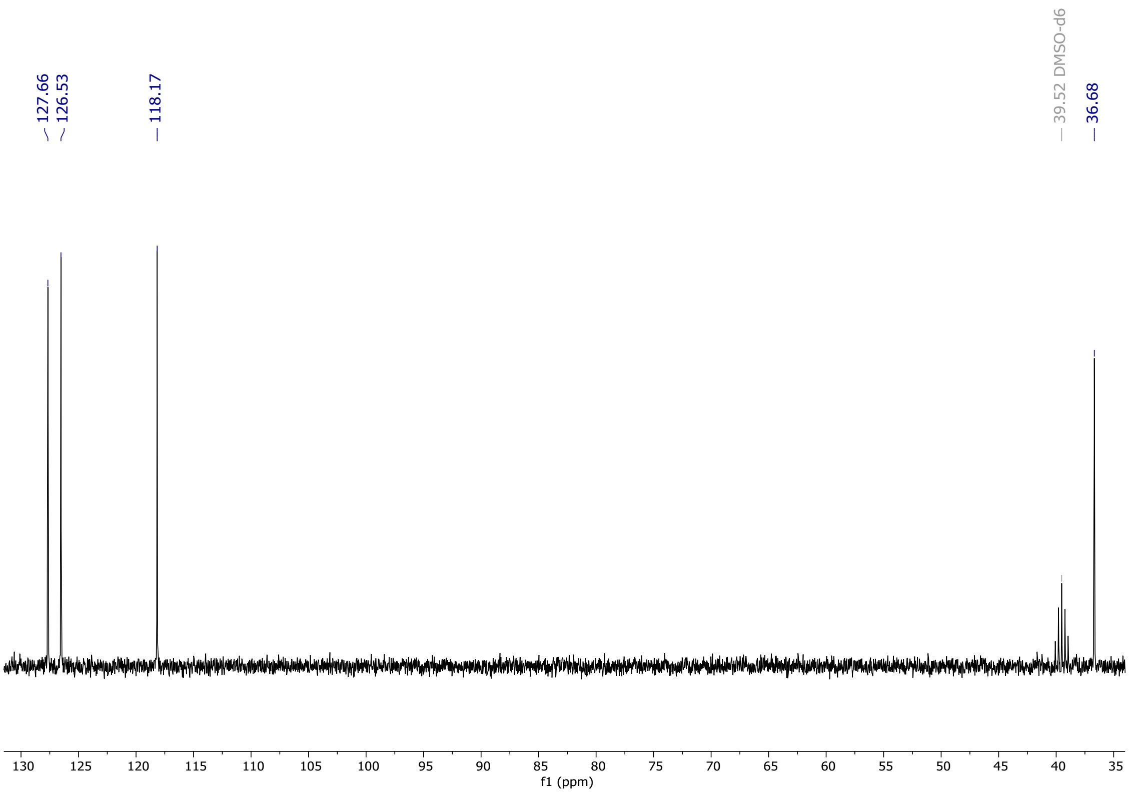

Figure S 4. ¹³C DEPT-135 of 10-methyl-3,7-dinitro-10H-phenothiazine

Synthesis of 10-methyl-10H-phenothiazine-3,7-diamine

In a 100mL round bottom flask, 1g (3.30 mmol, 1eq) of 10-methyl-3,7-dinitro-10H-phenothiazine was suspended in 50mL of ethanol. Then, and excess of $SnCl_2 \cdot 2H_2O$ (7g, 31.5 mmol) was added and the mixture refluxed vigorously for 8 hours. The obtained gray mixture has been pure into 500mL of water and basified with a saturated solution of sodium bicarbonate, until basic pH, paying attention of the possible gas release. The resulting mixture has been extracted with ethyl acetate three times with 200mL of solvent, the organic phases merged and dried over Na_2SO_4. The salt has been remove by filtration and the organic solvent by rotary evaporation. The resulted solid has been dissolved in 5mL of ethyl acetate and precipitated in 500mL of hexane. The resulting solid has been filtered with a Buckner funnel and wash abundantly with hexane. The resulting gray blueish solid has been dried in vacuum, to yield 0.8g (98%) of light blue fine powder. ^1H NMR (300 MHz, DMSO) δ 6.64 – 6.53 (m, 2H), 6.46 – 6.36 (m, 4H), 4.70 (s, 4H), 3.09 (bs, 3H). ^{13}C NMR (75 MHz, DMSO) δ 143.49, 136.49, 122.89, 114.34, 112.82, 112.59, 34.75.

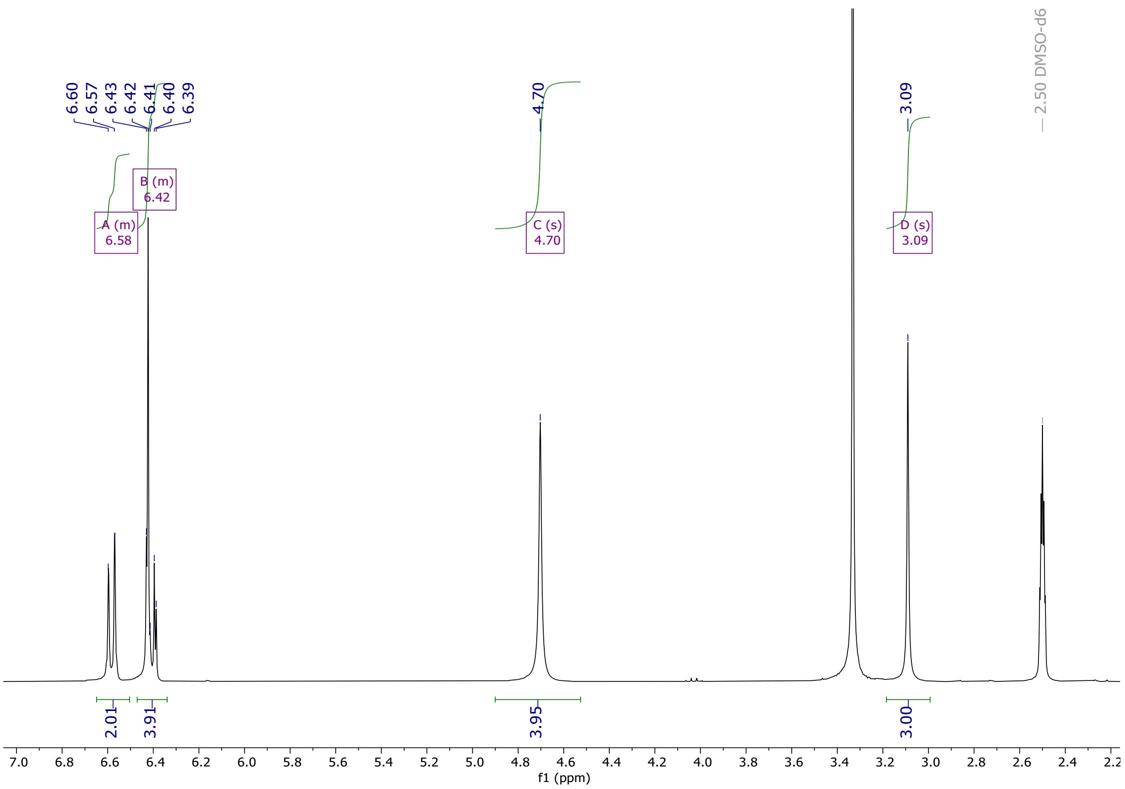

Figure S 5. ^{1}H NMR of 10-methyl-10H-phenothiazine-3,7-diamine

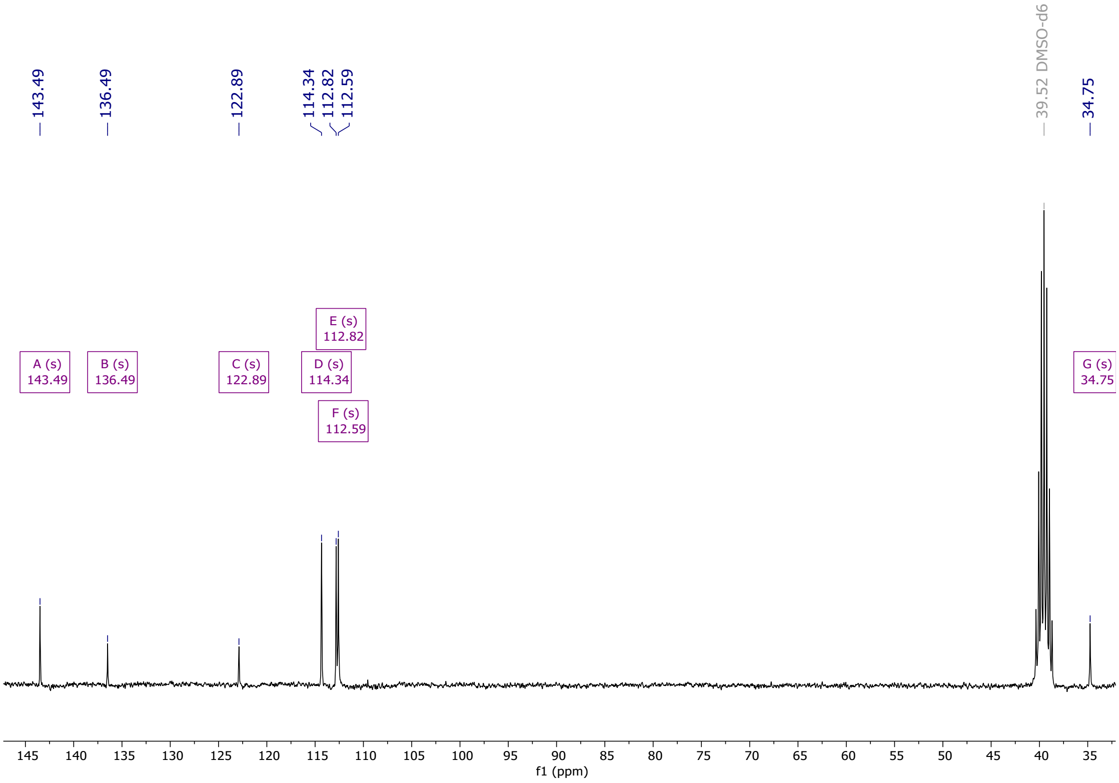

Figure S 6. 13C NMR of 10-methyl-10H-phenothiazine-3,7-diamine

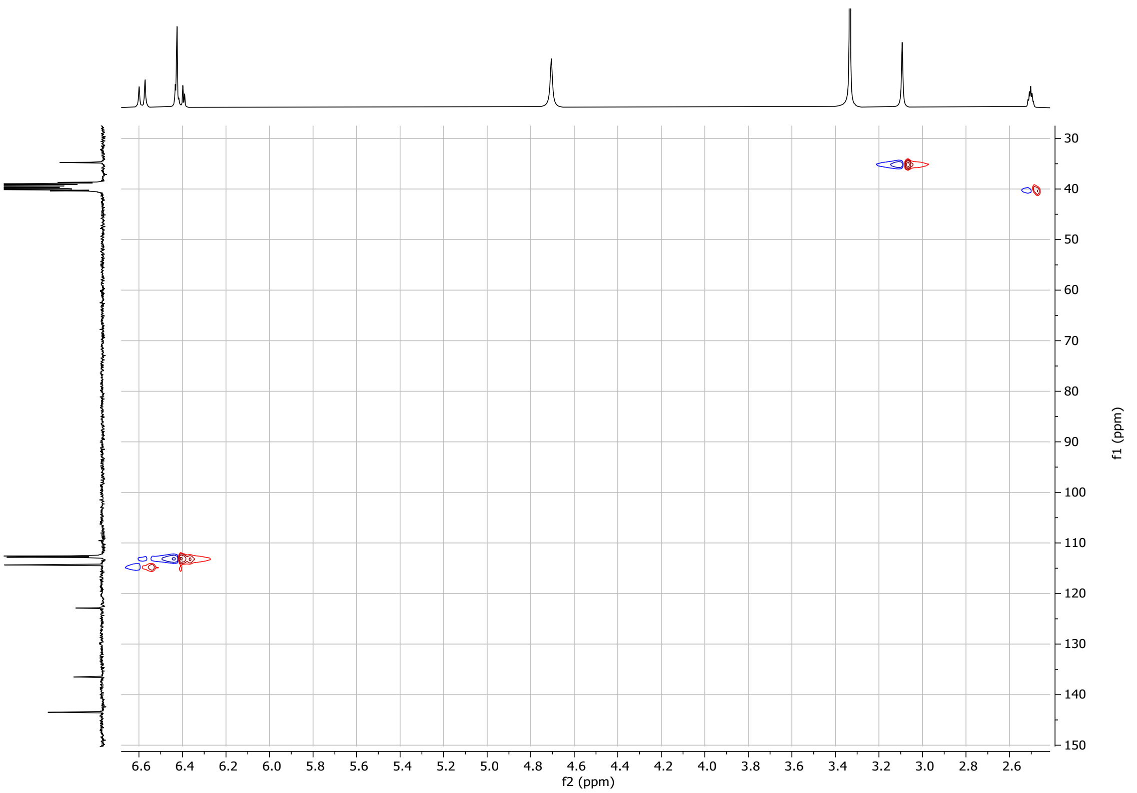

Figure S 7. ^{1}H-^{13}C HSQC of 10-methyl-10H-phenothiazine-3,7-diamine

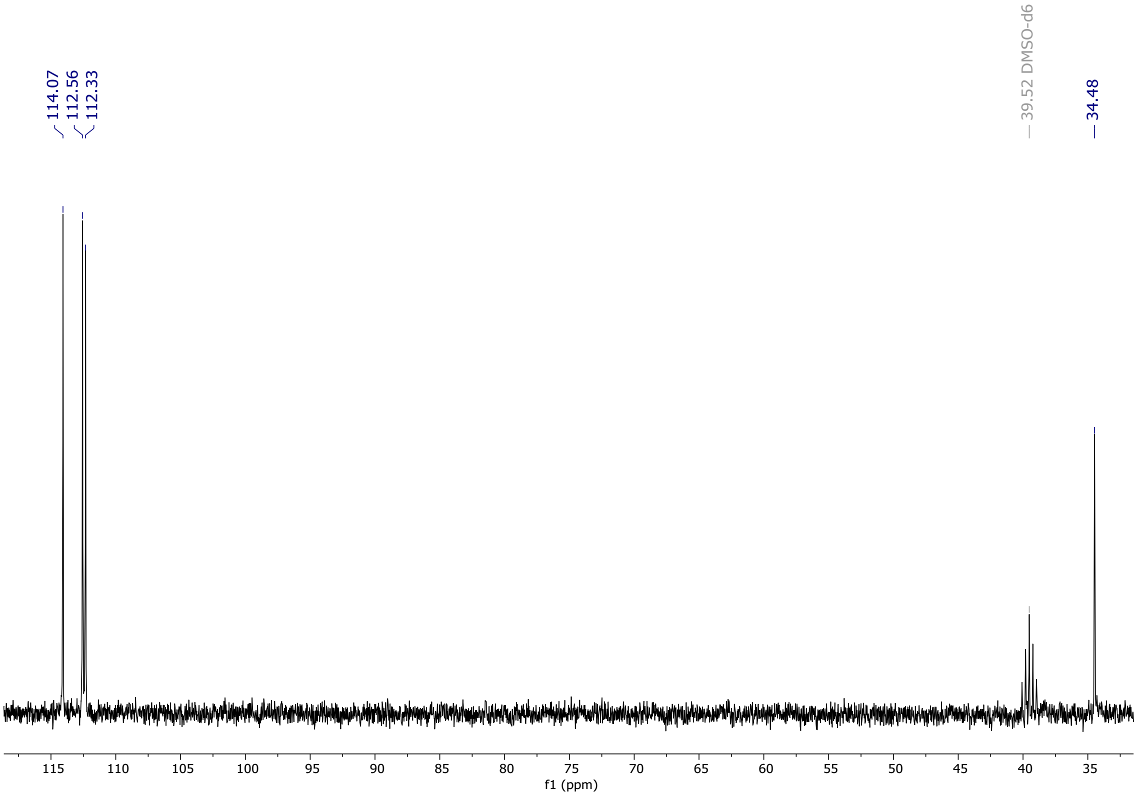

Figure S 8. 13C DEPT-135 of 10-methyl-10H-phenothiazine-3,7-diamine

Mellitic trianhydride (MTA) was synthesized as followed: Mellitic acid (1.05 g) was suspended in freshly distilled Ac_2O and heated to reflux under Argon atmosphere. The reaction mixture was refluxed for four hours. The green suspension was allowed to cool down to room temperature. Afterwards, the suspension was poured on dry Et_2O. The off-white precipitate (0.55 g, 52 %) was filtered and stored under Argon. ^{13}C NMR (100 MHz, DMSO-d_6, δ): 164.6, 162.7, 160.5, 158.1, 134.5, 134.0, 129.7 ppm.

Monomer salt synthesis

1 eq (128.04 mg, 0.3752 mmol) mellitic acid (MA) was dissolved in 40 mL deionized H_2O whilst stirring at room temperature. 1.5 eq (128.40 mg, 0.5261 mmol) phenothiazine was added in the solid state to the transparent MA-solution whilst vigorously stirring. The reaction mixture was heated up to 60 °C and stirred for approximately 4 hours. A green monomer salt (MS) powder was obtained, which was filtered over paper (pore size 5-13 µm) and dried overnight at 55 °C in an oven.

Synthesis of dual redox-active porous polyimide

DA-MPT (1.5 eq., 0.3 mmol) and MTA (1 eq., 0.2 mmol) were weighed into a microwave reactor (Anton Paar, G30) and suspended in 10 mL degassed H_2O. After adding a stirring bar, the reactor was closed with a cap including a PTFE septum and placed in the Anton Paar Monowave 400. The vessel was heated as fast as possible to 180 °C, stirred with 600 rpm, and held at that temperature for 1 h. Afterwards, the reactor was cooled down to 70 °C and the dark blue precipitate was collected via filtration. The product was washed using a soxhlet extraction method with tetrahydrofuran to remove unreacted monomers and small oligomers. Finally, the product (dark blue powder, 104 mg) was dried overnight, in vacuo, at 80 °C.

Result and discussions

Elemental analysis

Species 1: decarboxylation of two moieties – percolated polymer network

Calculated formula: $C_{59}H_{27}N_9O_8S_3$

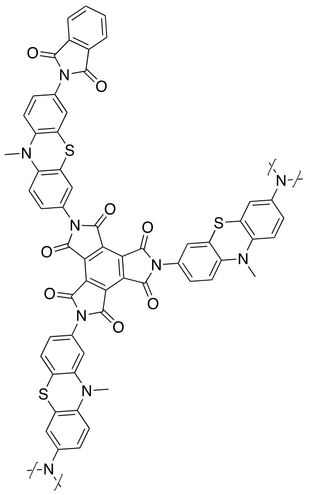

Calculated C/N ratio: 6.556

Species 2: one amide moiety

Calculated formula: $C_{63}H_{29}N_{11}O_{13}S_3$

Calculated C/N ratio: 5.727

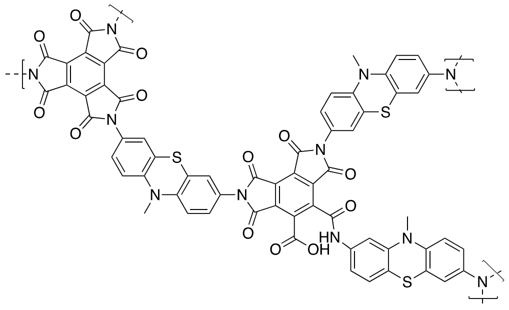

Sample name	Experimental C %	Experimental N %	Experimental H %	Experimental molar ratio C/H	N/H	C/N
ML215	55,6	9,72	3,78	1,234	0,185	6,671
	55,97	10,07	3,89	1,207	0,186	6,482
Average:	55,785	9,895	3,835	1,221	0,186	6,575
ML201	59,62	10,8	3,73	1,341	0,208	6,438
	59,41	10,63	3,68	1,355	0,208	6,518
Average:	59,515	10,715	3,705	1,348	0,208	6,477

Table S 1. Elemental analysis of the MTA-MTP porous polyimides

Pore size distribution and hypothesis for pore sizes

The PSD of PI(MTA MPT) is displayed below, evincing two populations around at ca. 0.5 nm and 0.8 nm:

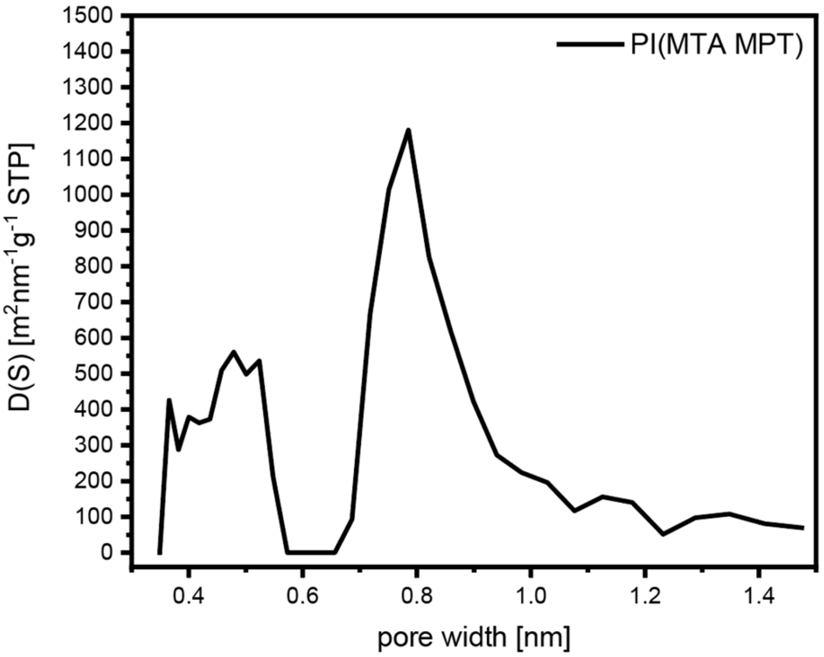

Figure S 9. PSD of MTA-MPT porous polyimide

Displayed below is a possible theoretical structure of a pore of a fully condensed PI(MTA MPT) network (A: top view; B: side view). From PXRD we know that PI(MTA-MPT) is not crystalline, but rather semicrystalline. Hence, significant disordered such as at aryl-N_{imide} axes is expected (indicated by red arrows). Furthermore, the phenothiazine moiety is not planar, but kinked (see C: shown is an excerpt from the crystal structure of a *N*-ethyl-phenothiazine containing Ni complex, extracted from the Cambridge crystallographic data centre CCDC, Refcode: 700928). For both the expected aryl-N axis disorder and the kinked structure of the phenothiazine segment, the network is expected to not be planar but "wavy"/kinked. As displayed in D: The N-N distance (d_{N-N}) in the phenothiazine moiety can be calculated as 9.981 Å (hypothesis: The distance corresponds roughly to the C3-C30 distance in CCDC Refcode: 700928). We expect N-N distance of the mellitic imide subunit to correspond to the O-O distance of mellitic trianhydride (extracted from CCDC Refcode 135999), i.e., 5.907 Å. Fixing the angle Cat 120 °(which would correspond to a flat pore), we obtain a pore diameter of ca. c= 1.4 nm. For an

interpenetrated structure – such as shown in E – two pore size populations at around 0.5 nm and 0.8 nm fit relatively well.

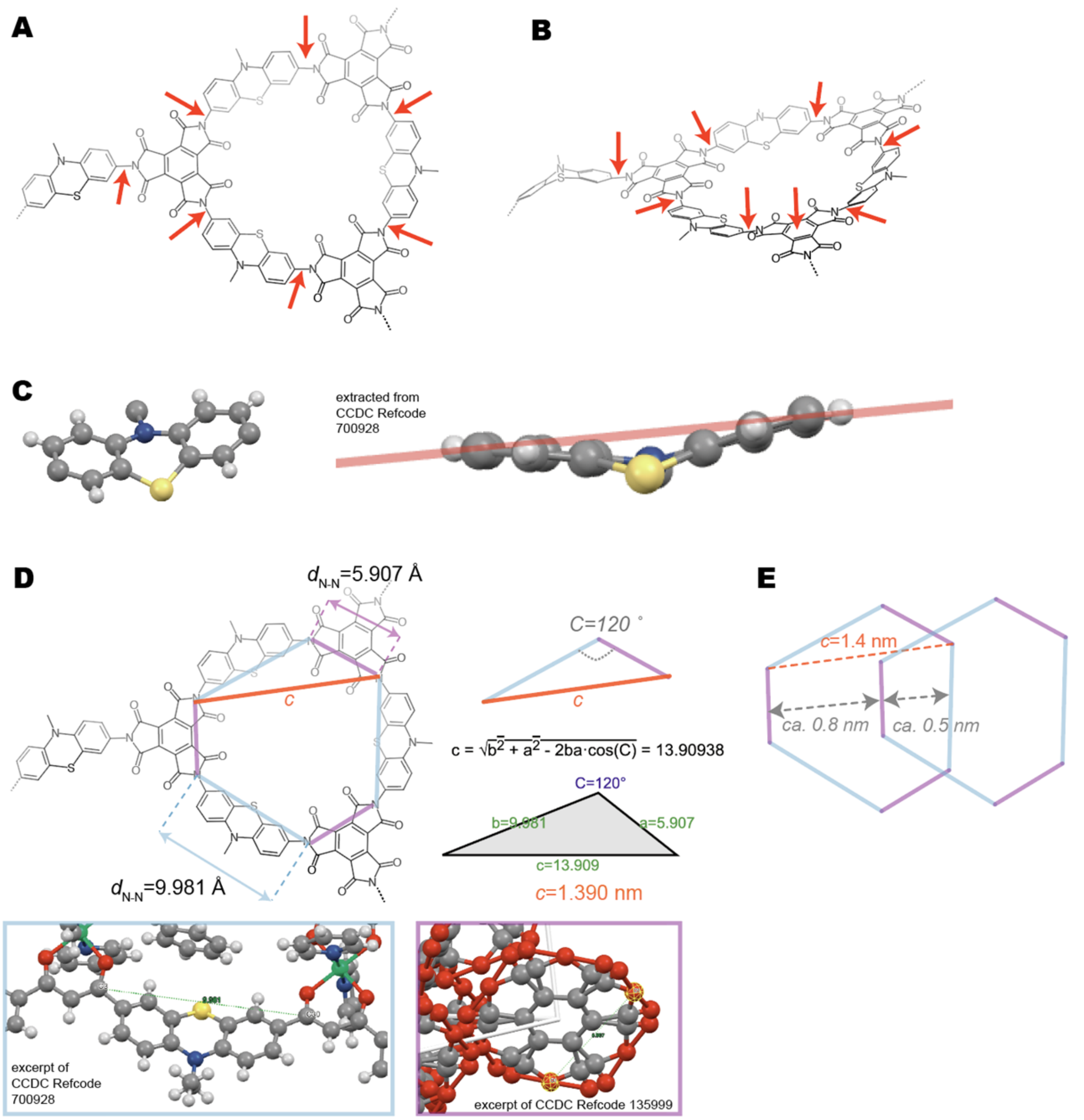

Figure S 10. (a) Top and (b) side view of the theoretical structure of a pore of a fully condensed PI(MTA MPT) network. (c) Expected crystal structure of a N-ethyl-phenothiazine containing Ni complex. Pore size calculations for a single and interpenetrated structure

SEM images

Below are additional SEM image of the MTA-MPT porous polyimide, as synthesized.

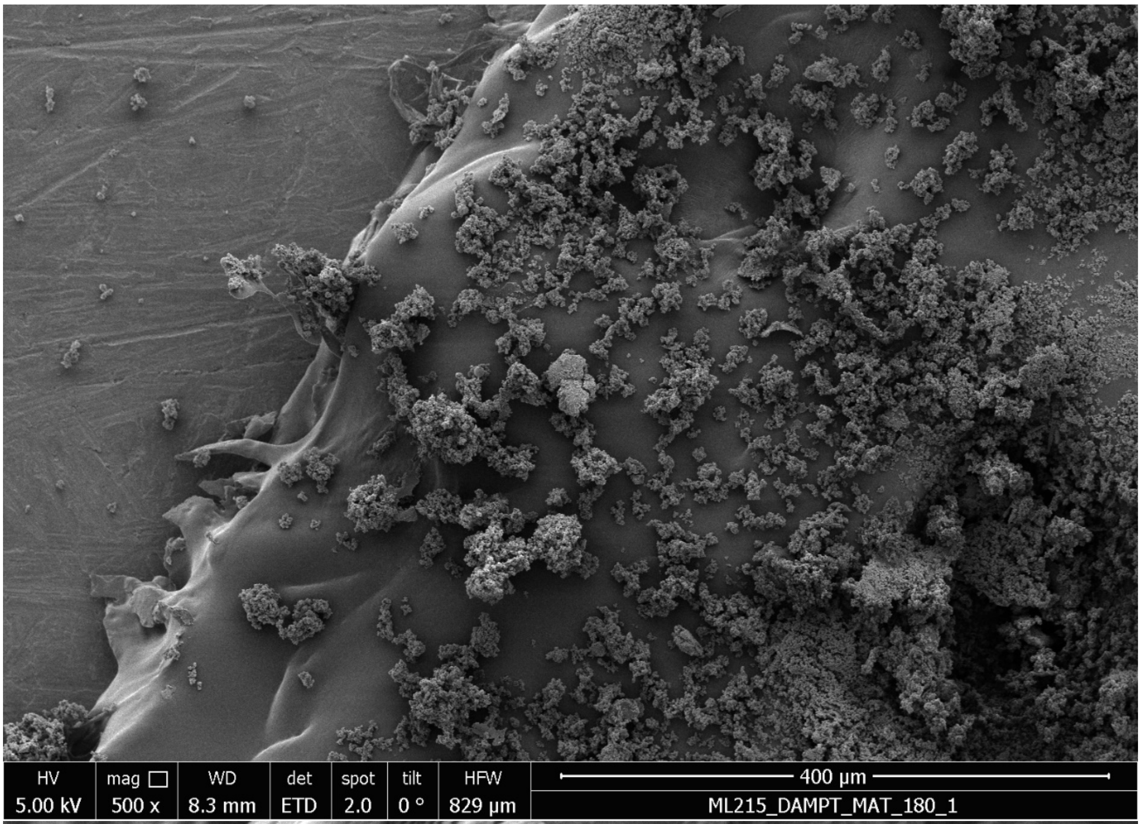

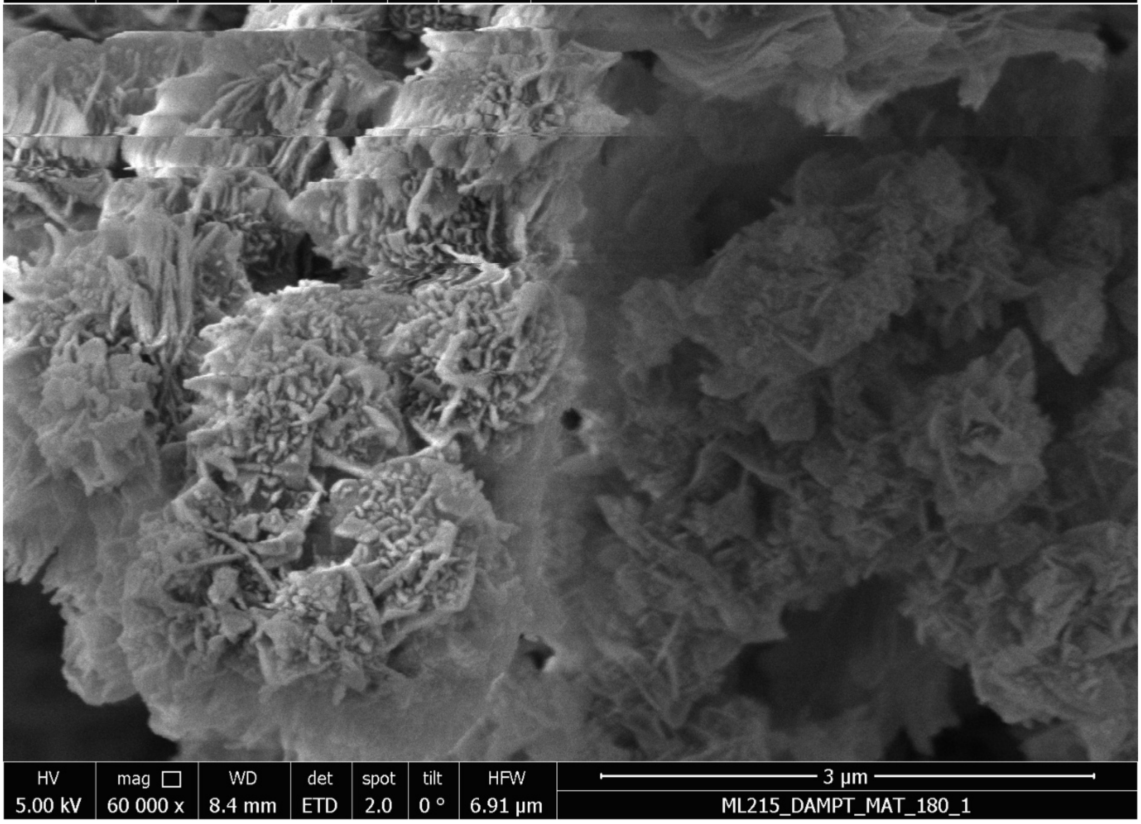

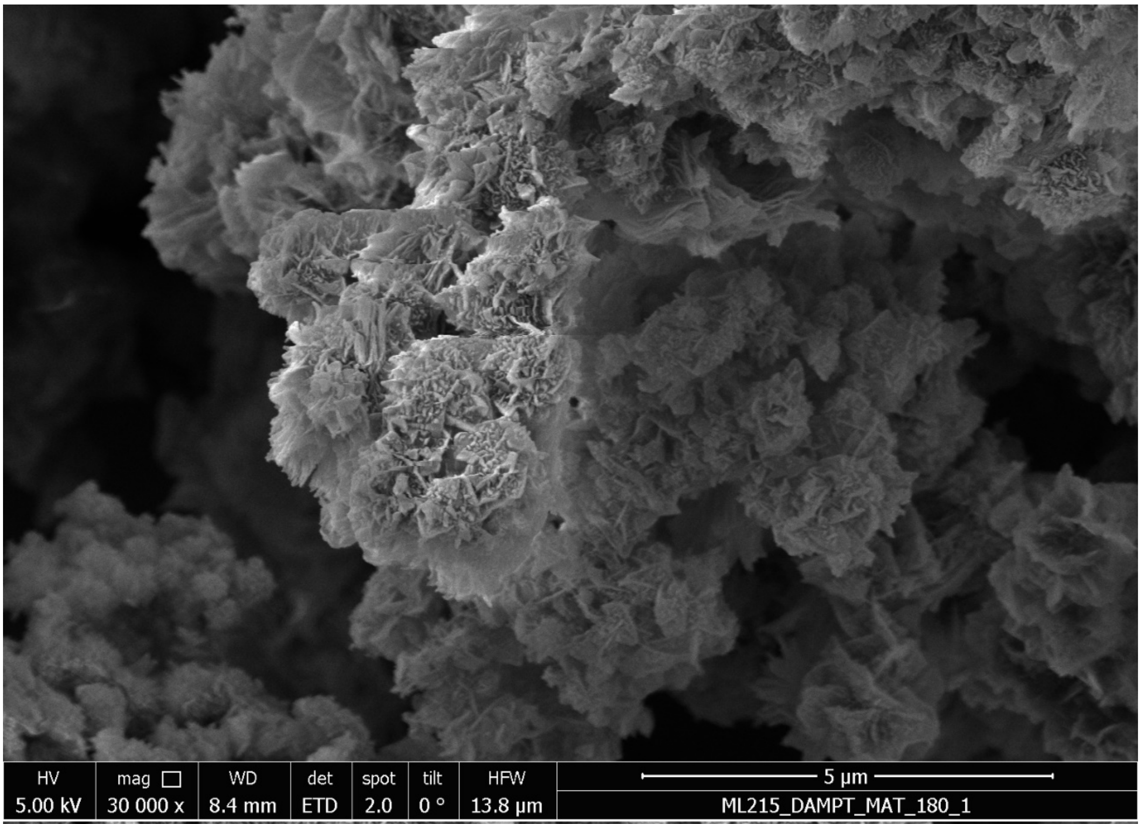
HV
5.00 kV
mag
30 000 x
WD
8.4 mm
det
ETD
spot
2.0
tilt
0 °
HFW
13.8 µm
5 µm
ML215_DAMPT_MAT_180_1

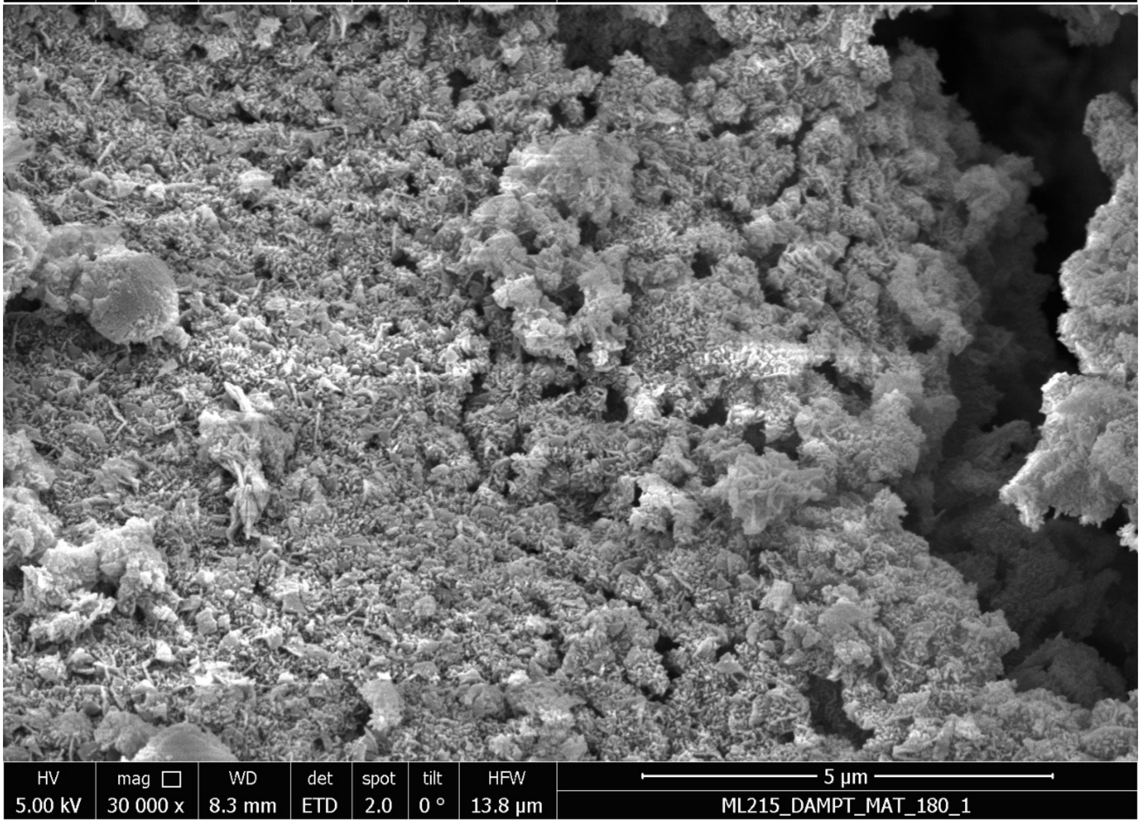
HV
5.00 kV
mag
30 000 x
WD
8.3 mm
det
ETD
spot
2.0
tilt
0 °
HFW
13.8 µm
5 µm
ML215_DAMPT_MAT_180_1

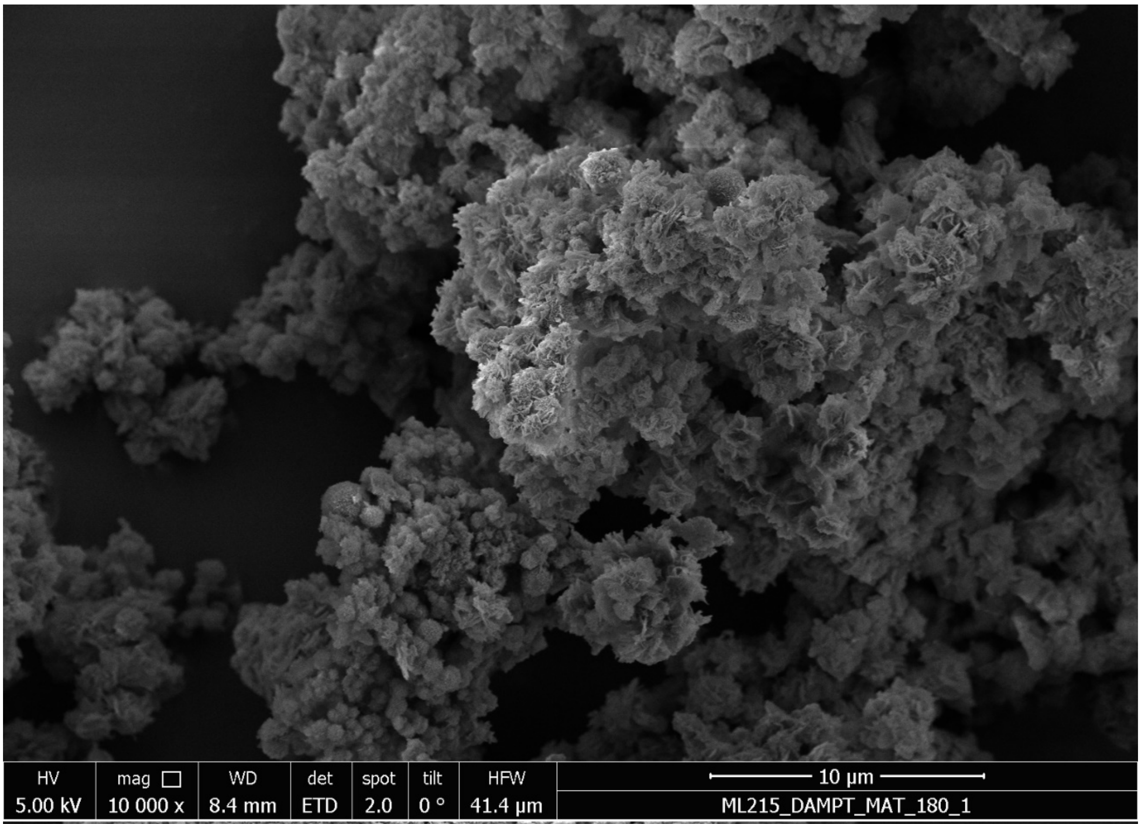
HV
5.00 kV
mag
10 000 x
WD
8.4 mm
det
ETD
spot
2.0
tilt
0 °
HFW
41.4 µm
10 µm
ML215_DAMPT_MAT_180_1

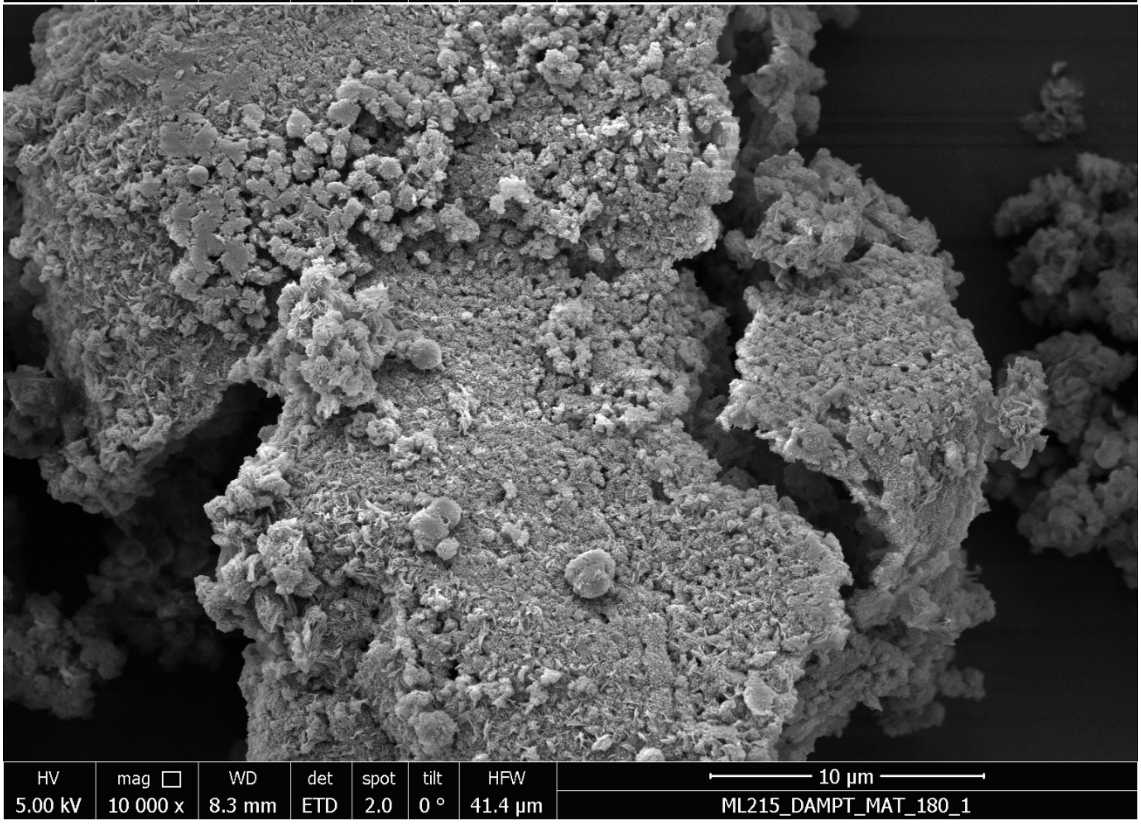
HV
5.00 kV
mag
10 000 x
WD
8.3 mm
det
ETD
spot
2.0
tilt
0 °
HFW
41.4 µm
10 µm
ML215_DAMPT_MAT_180_1

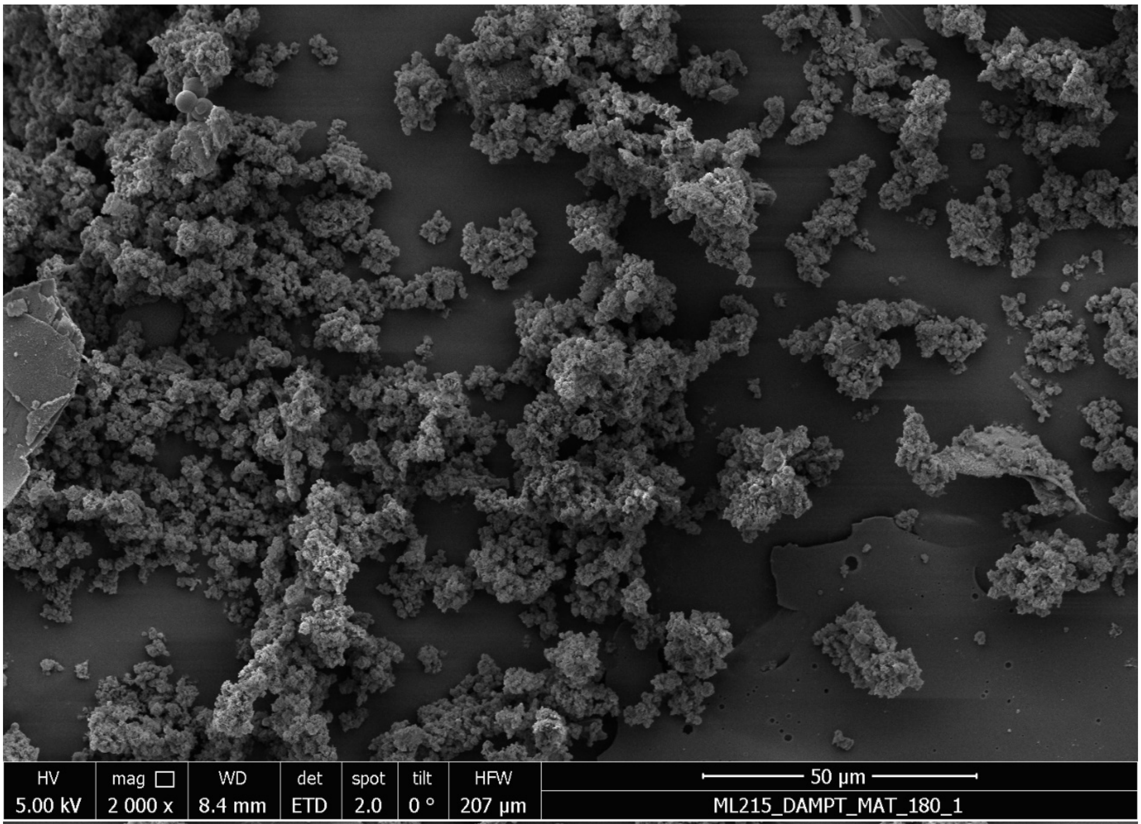
HV
5.00 kV
mag
2 000 x
WD
8.4 mm
det
ETD
spot
2.0
tilt
0 °
HFW
207 µm
50 µm
ML215_DAMPT_MAT_180_1

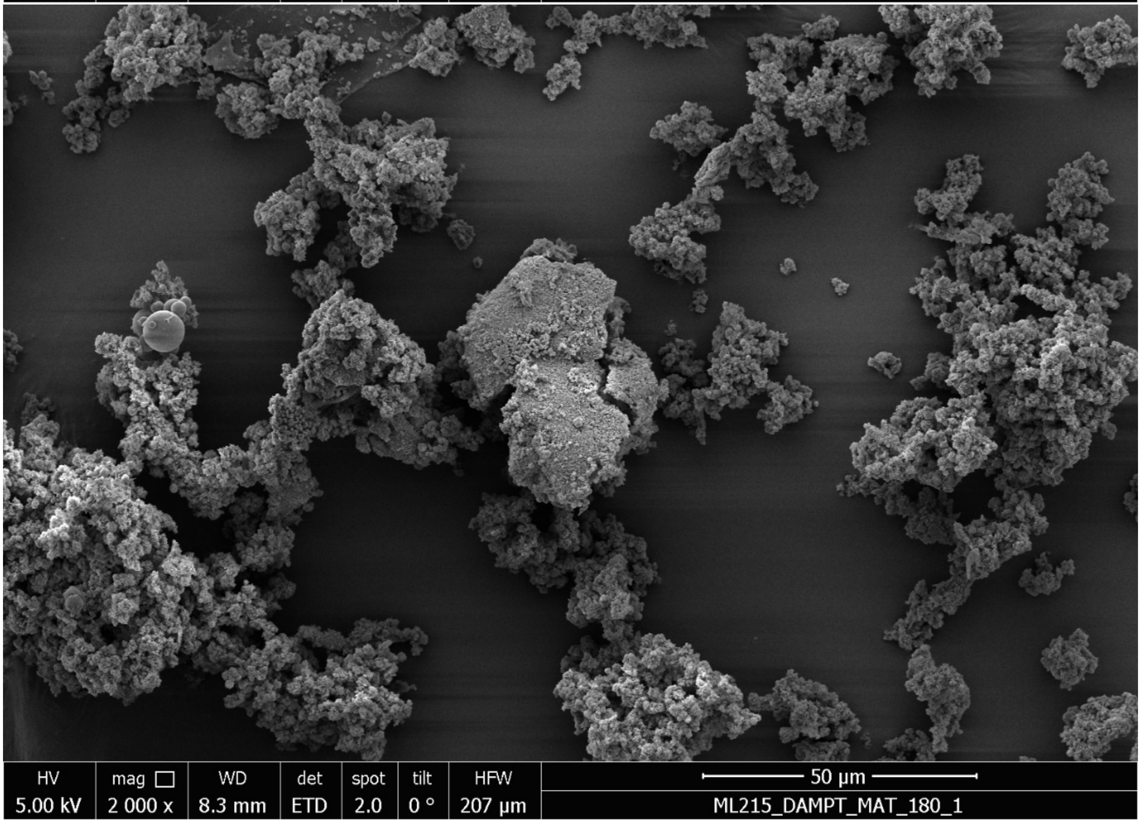
HV
5.00 kV
mag
2 000 x
WD
8.3 mm
det
ETD
spot
2.0
tilt
0 °
HFW
207 µm
50 µm
ML215_DAMPT_MAT_180_1

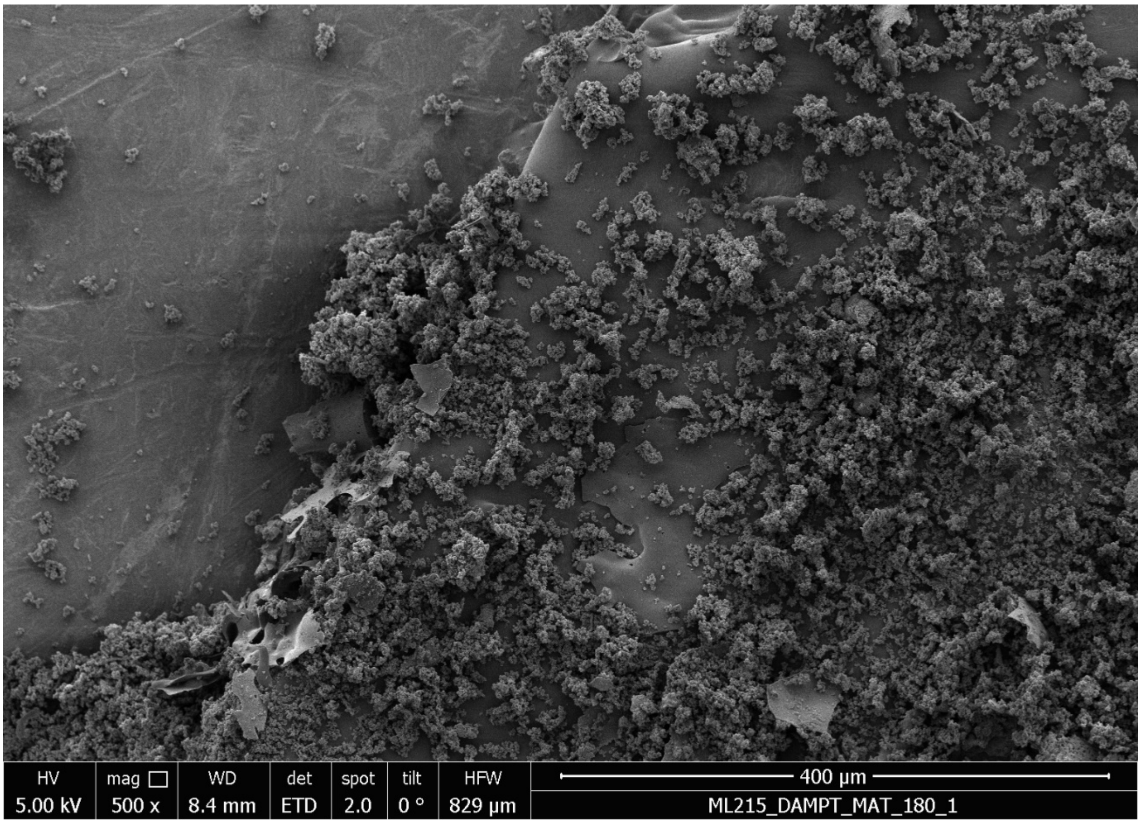
HV
5.00 kV
mag
500 x
WD
8.4 mm
det
ETD
spot
2.0
tilt
0 °
HFW
829 µm
400 µm
ML215_DAMPT_MAT_180_1

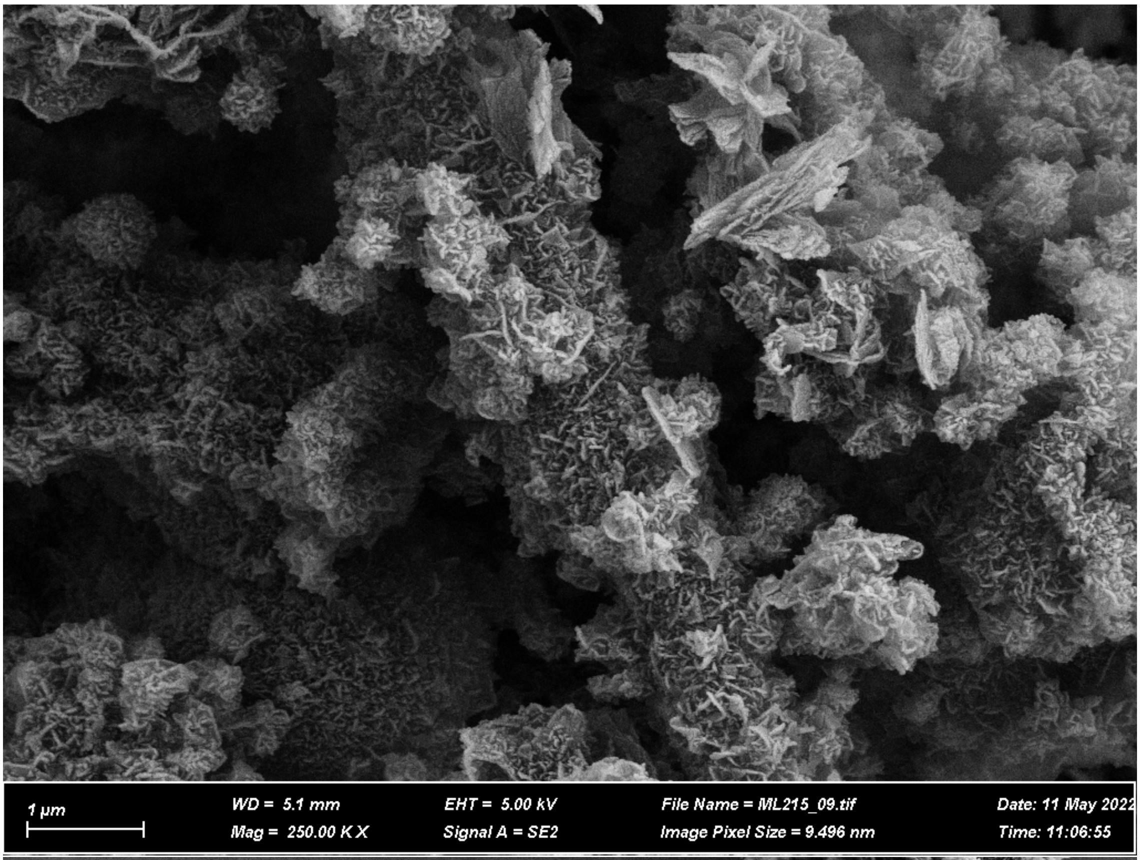
1 µm
WD = 5.1 mm
Mag = 250.00 K X
EHT = 5.00 kV
Signal A = SE2
File Name = ML215_09.tif
Image Pixel Size = 9.496 nm
Date: 11 May 2022
Time: 11:06:55

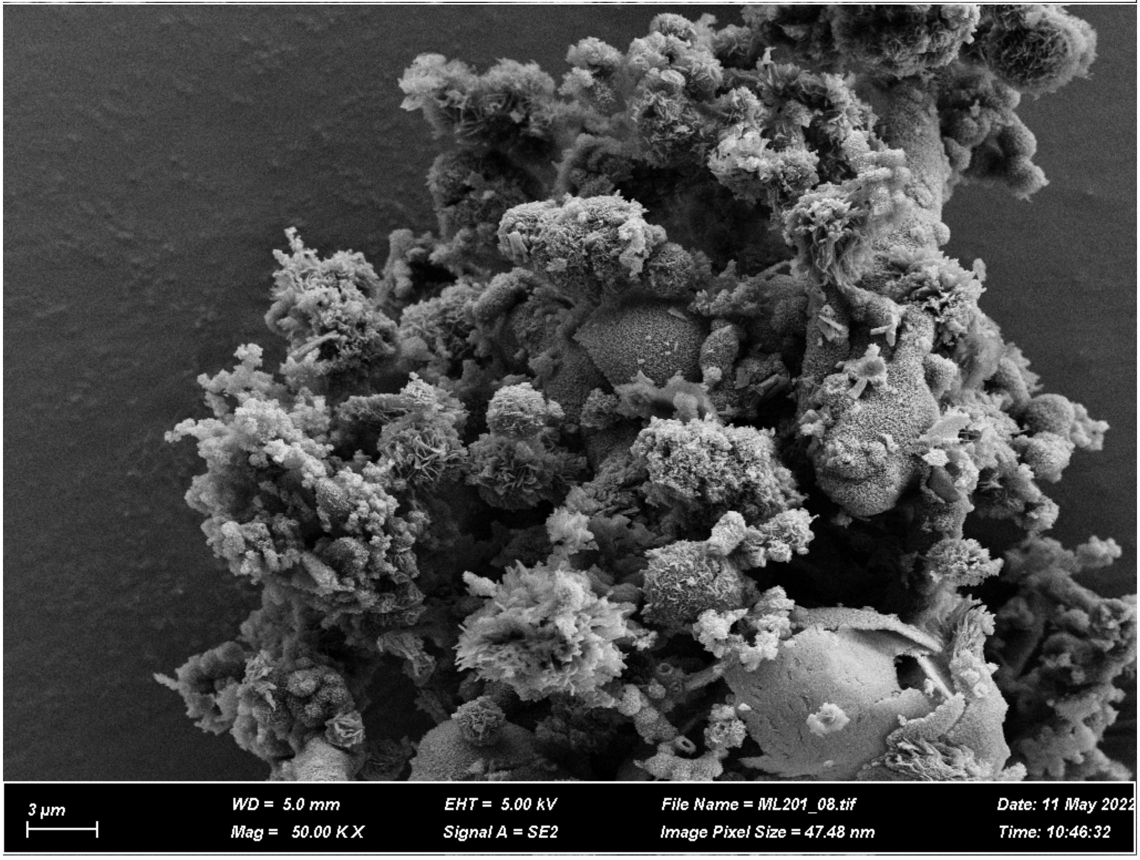
3 µm
WD = 5.0 mm
Mag = 50.00 K X
EHT = 5.00 kV
Signal A = SE2
File Name = ML201_08.tif
Image Pixel Size = 47.48 nm
Date: 11 May 2022
Time: 10:46:32

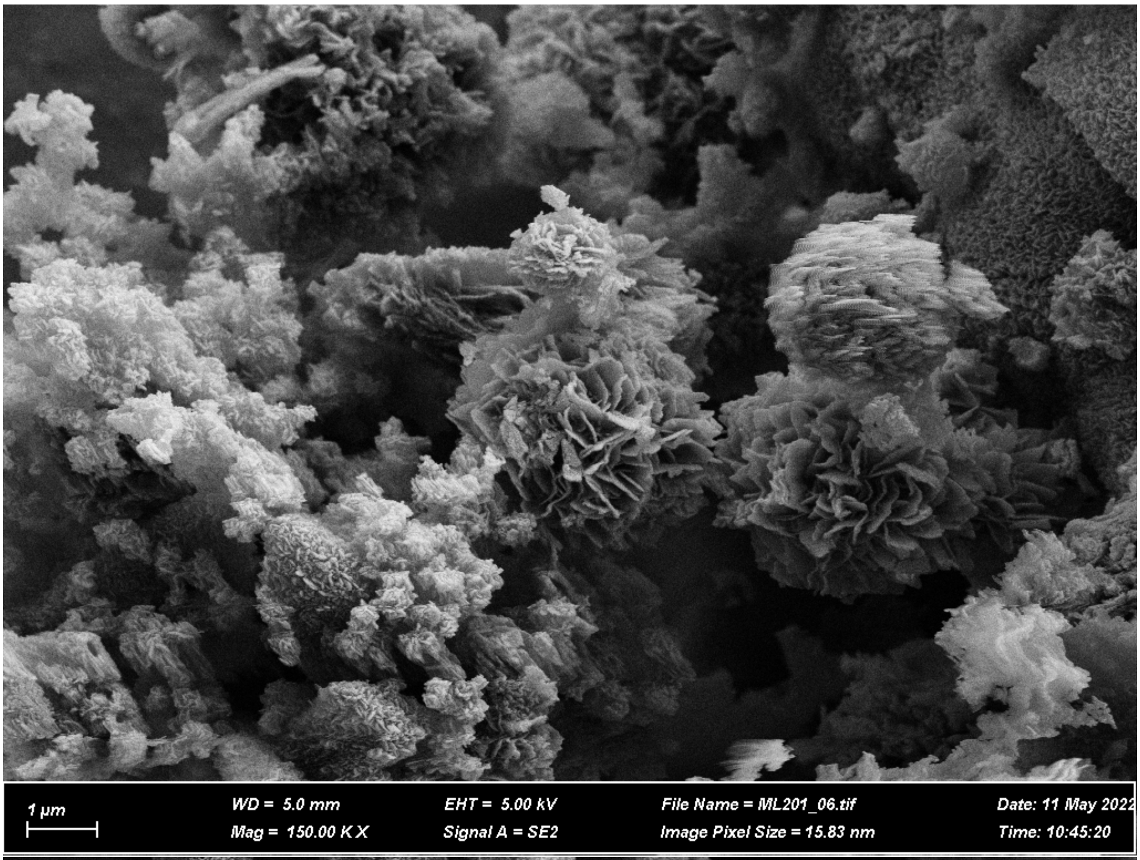
1 µm
WD = 5.0 mm
Mag = 150.00 K X
EHT = 5.00 kV
Signal A = SE2
File Name = ML201_06.tif
Image Pixel Size = 15.83 nm
Date: 11 May 2022
Time: 10:45:20

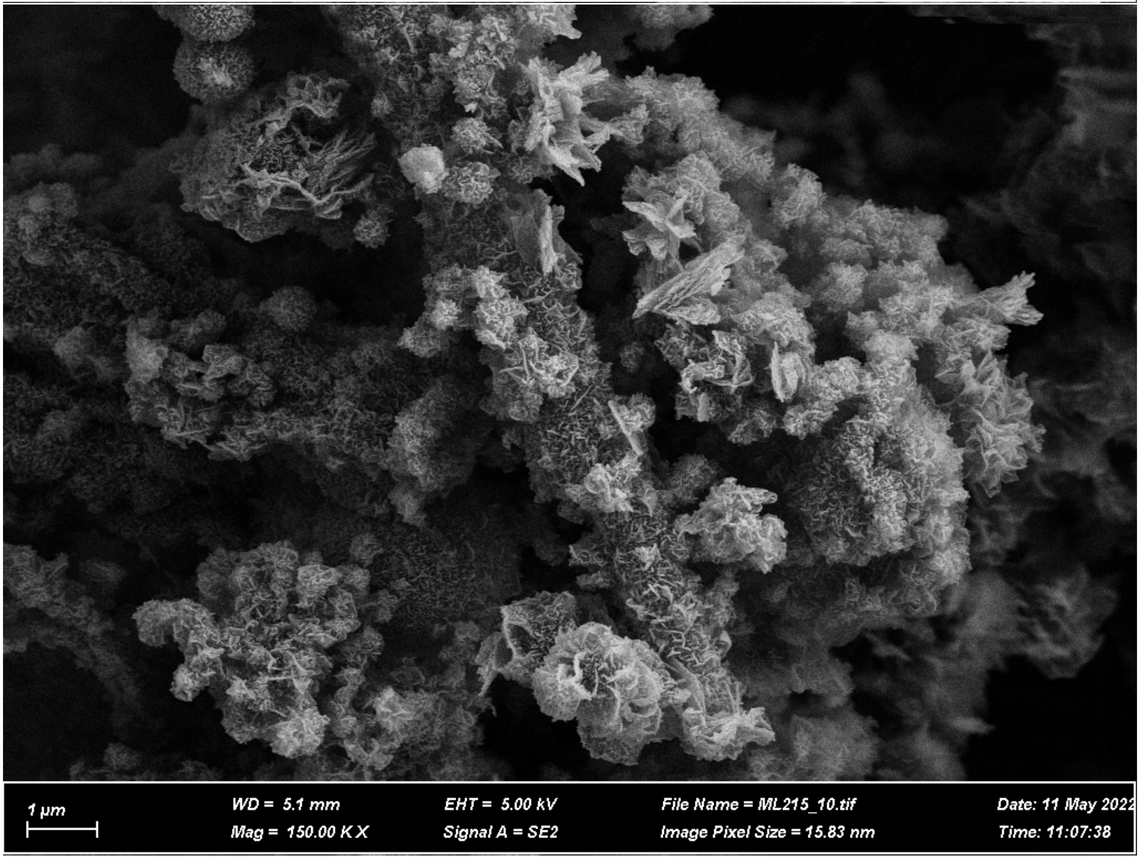
1 µm
WD = 5.1 mm
Mag = 150.00 K X
EHT = 5.00 kV
Signal A = SE2
File Name = ML215_10.tif
Image Pixel Size = 15.83 nm
Date: 11 May 2022
Time: 11:07:38

lithium metal │MTA-MPT porous PI battery cycling

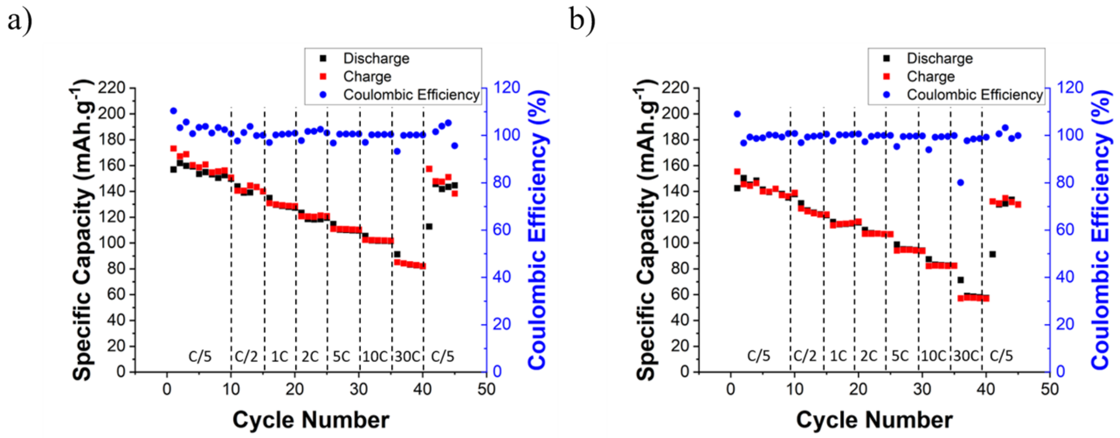

Figure S 11. Galvanostatic cycling of the MTA-MPT porous polyimide in a lithium metal battery at various C-rate with a voltage ranges of 1.5 V ─3.8 V (both imide & phenothiazine processes) using either (a) 1 M LiTFSI DOL/DME or (b) 1 M $LiPF_6$ EC/DEC as electrolyte.

References

1. Thommes, M. *et al.* Physisorption of gases, with special reference to the evaluation of surface area and pore size distribution (IUPAC Technical Report). *Pure Appl. Chem.* **87**, 1051–1069 (2015).

2. Guilmin, R., Alloin, F., Molton, F. & Leprêtre, J. C. Chemical modification of N-methylphenothiazine to lead to interesting and potential organic material for lithium battery. *Electrochim. Acta* **232**, 182–191 (2017).
